# The Efficacy of the Interferon Alpha/Beta Response versus Arboviruses Is Temperature Dependent

**DOI:** 10.1128/mBio.00535-18

**Published:** 2018-04-24

**Authors:** Whitney C. Lane, Matthew D. Dunn, Christina L. Gardner, L. K. Metthew Lam, Alan M. Watson, Amy L. Hartman, Kate D. Ryman, William B. Klimstra

**Affiliations:** a Center for Vaccine Research, University of Pittsburgh, Pittsburgh, Pennsylvania, USA; b Department of Immunology, University of Pittsburgh, Pittsburgh, Pennsylvania, USA; c Department of Infectious Disease and Microbiology, University of Pittsburgh, Pittsburgh, Pennsylvania, USA; d Department of Cell Biology, University of Pittsburgh, Pittsburgh, Pennsylvania, USA; e Department of Microbiology and Molecular Genetics, University of Pittsburgh, Pittsburgh, Pennsylvania, USA; Brown University

**Keywords:** alphavirus, arbovirus, chikungunya virus, interferons, temperature

## Abstract

Interferon alpha/beta (IFN-α/β) is a critical mediator of protection against most viruses, with host survival frequently impossible in its absence. Many studies have investigated the pathways involved in the induction of IFN-α/β after virus infection and the resultant upregulation of antiviral IFN-stimulated genes (ISGs) through IFN-α/β receptor complex signaling. However, other than examining the effects of genetic deletion of induction or effector pathway components, little is known regarding the functionality of these responses in intact hosts and whether host genetic or environmental factors might influence their potency. Here, we demonstrate that the IFN-α/β response against multiple arthropod-vectored viruses, which replicate over a wide temperature range, is extremely sensitive to fluctuations in temperature, exhibiting reduced antiviral efficacy at subnormal cellular temperatures and increased efficacy at supranormal temperatures. The effect involves both IFN-α/β and ISG upregulation pathways with a major aspect of altered potency reflecting highly temperature-dependent transcription of IFN response genes that leads to altered IFN-α/β and ISG protein levels. Discordantly, signaling steps prior to transcription that were examined showed the opposite effect from gene transcription, with potentiation at low temperature and inhibition at high temperature. Finally, we demonstrate that by lowering the temperature of mice, chikungunya arbovirus replication and disease are exacerbated in an IFN-α/β-dependent manner. This finding raises the potential for use of hyperthermia as a therapeutic modality for viral infections and in other contexts such as antitumor therapy. The increased IFN-α/β efficacy at high temperatures may also reflect an innate immune-relevant aspect of the febrile response.

## IMPORTANCE

The interferon alpha/beta (IFN-α/β) response is a first-line innate defense against arthropod-borne viruses (arboviruses). Arboviruses, such as chikungunya virus (CHIKV), can infect cells and replicate across a wide temperature range due to their replication in both mammalian/avian and arthropod hosts. Accordingly, these viruses can cause human disease in tissues regularly exposed to temperatures below the normal mammalian core temperature, 37°C. We questioned whether temperature variation could affect the efficacy of IFN-α/β responses against these viruses and help to explain some aspects of human disease manifestations. We observed that IFN-α/β efficacy was dramatically lower at subnormal temperatures and modestly enhanced at febrile temperatures, with the effects involving altered IFN-α/β response gene transcription but not IFN-α/β pathway signaling. These results provide insight into the functioning of the IFN-α/β response *in vivo* and suggest that temperature elevation may represent an immune-enhancing therapeutic modality for a wide variety of IFN-α/β-sensitive infections and pathologies.

## INTRODUCTION

Type I interferon (IFN) is a critical early protector of vertebrate hosts from overwhelming viral replication and disease (reviewed in reference 1). This role has been abundantly demonstrated with arboviruses and other viruses by infection of *Ifnar1*^−/−^ mice that lack interferon alpha/beta (IFN-α/β) signaling. In many cases, a completely benign localized infection of normal mice is rendered systemic and rapidly fatal by elimination of signaling through the IFN-α/β receptor (e.g., see references 2 to 5). Even minor changes in the characteristics of the IFN-α/β response greatly increase viral replication and disease (6).

The IFN-α/β response consists of an inductive phase in which virus infection stimulates infected and possibly uninfected cells to produce and secrete IFN-α and IFN-β proteins that signal through the dimeric IFN-α/β receptor and cause the transcriptional upregulation of antiviral effector genes (7, 8). This leads to production of proteins in infected and uninfected cells that, together, constitute an “antiviral state.” This response is the primary protector of vertebrate hosts from overwhelming virus replication prior to development of the adaptive immune response and is also involved in the clearance phase of infection (9, 10). Subclinical infections with arboviruses and other viruses that normally affect only regional tissues can be rendered systemic and catastrophic by the absence of this response (2, 3, 11). The type I IFN response is so effective that it can determine apparent tissue tropism for highly IFN-α/β-sensitive viruses (2). This suggests that even minor increases or decreases in the efficacy of this response could have dramatic impacts upon the outcome of virus infection.

Previous studies have primarily focused upon the characteristics of IFN-α/β induction and effector phases under standard laboratory conditions mimicking mammalian core temperatures (e.g., 37°C). However, temperatures in peripheral tissues of humans can range as much as 5°C below 37°C under normal room-temperature conditions (12) and can become much lower under more extreme conditions (13). In addition, few studies have examined IFN-α/β responses under the febrile conditions under which they commonly act *in vivo* during pathogen infection. Indeed, core (rectal) temperatures can rise to 42°C during extreme febrile or hyperpyrexic episodes, and febrile responses to infection typically range between 38 and 40°C (14). However, two recent studies have suggested that IFN-α/β responses may be lesser in upper airway epithelia, where temperatures are substantially below core (15, 16). This work complemented several historical studies that also suggested temperature-mediated effects on IFN-α/β efficacy in other model systems (17–23). Other early studies, however, including some with arboviruses, which identified temperature variation as a factor in IFN-α/β effectiveness implicated temperature-altered virus replication rather than the IFN-α/β response (24–35).

Here, we demonstrate that multiple arboviruses, including alphaviruses, bunyaviruses, and flaviviruses, which have evolved to replicate at ambient temperatures in the invertebrate vector, are dramatically more resistant to the IFN-α/β system at temperatures below 37°C. Subnormal temperatures led to a considerable diminution of IFN-α/β efficacy, and supranormal temperatures led to a modest enhancement of efficacy, possibly representing an evolutionary mechanism for the febrile response. At the same time, induction of IFN-α/β was delayed and reduced at low temperatures and enhanced at higher temperatures *in vitro*. A primary mechanism underlying the temperature effect appeared to involve reduced rates of gene transcription at the lower temperatures. Furthermore, in a mouse model of chikungunya virus (CHIKV) infection and musculoskeletal disease (MSD), lowered temperatures induced in torpid or reserpine-treated mice resulted in exacerbation of virus replication and signs of MSD in an IFN-α/β response-dependent manner. The results of these studies will have impacts upon the understanding of pathogenesis of all arboviruses as well as other viruses that replicate in sites with altered temperature (e.g., rhinovirus [RV], influenza virus, and coronavirus) as well as other therapeutic contexts in which IFN is used (e.g., oncology). This may lead to improved therapeutic modalities involving localized or systemic temperature modification.

## RESULTS

### Efficacy of IFN-α/β against arboviruses is reduced at subnormal temperatures.

**FIG 1:**
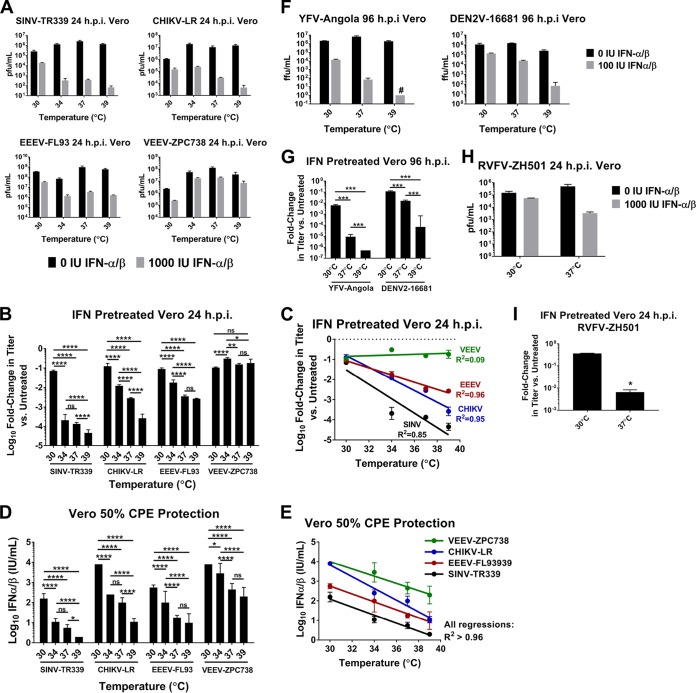
Efficacy of type I IFN against arboviruses is reduced at subnormal temperatures. (A to C) Vero cells were treated overnight with 0 or 1,000 IU IFN-α/β at 30, 34, 37, or 39°C and infected with the indicated alphaviruses at an MOI of 0.1. Supernatants were collected at 24 hpi, and viral titers were determined by plaque assay on BHK cells at 37°C. (A) Comparison of viral growth at 24 hpi between temperatures with and without IFN-α/β treatment. (B) Log_10_ fold change in viral titer between IFN-α/β-treated and untreated cells at each temperature ± SD. *, *P* < 0.05; **, *P* < 0.01; ***, *P* < 0.001; ****, *P* < 0.0001; ns, not significant by two-way analysis of variance with Tukey’s multiple-comparison test on log-transformed fold change values. (C) Significant linear correlation between increasing temperature and viral growth inhibition by IFN-α/β pretreatment for IFN-α/β-sensitive alphaviruses. Pearson’s correlation, *P* < 0.02 for EEEV and CHIKV; *P* < 0.07 for SINV. (D and E) EC_50_ of IFN-α/β in Vero cells at 30, 34, 37, and 39°C against the indicated alphaviruses was determined by IFN-α/β bioassay. (D) Data are presented as log_10_ mean IU per milliliter required to protect 50% of Vero cells from virus-induced cytopathic effect at each temperature ± SD. *, *P* < 0.05; **, *P* < 0.01; ***, *P* < 0.001; ****, *P* < 0.0001; ns, not significant by two-way analysis of variance with Tukey’s multiple-comparison test on log-transformed IU/milliliter values. (E) Significant linear correlation between increasing temperature and decreasing IFN-α/β EC_50_ was established using Pearson’s correlation on log-transformed IU/milliliter values (*P* < 0.02 for all viruses). (F and G) Vero cells were treated overnight with 0 or 100 IU IFN-α/β at 30, 37, or 39°C and infected with YFV or DENV at an MOI of 0.1. At 96 hpi, supernatants were assayed for viral titer by focus-forming assay at 37°C. (F) Comparison of viral growth at 96 hpi between temperatures with and without IFN-α/β treatment. (G) Data are expressed as mean fold change in titer between IFN-α/β-primed and unprimed cells at each temperature ± SD. ***, *P* < 0.001 two-way analysis of variance with Tukey’s multiple-comparison test of log-transformed fold change values. (H and I) The procedure from panel A was repeated with RVFV at an MOI of 5 to 10, using only 30 and 37°C temperature conditions. At 24 hpi, supernatants were assayed for viral titer by plaque assay. (H) Comparison of viral growth at 24 hpi between temperatures with and without IFN-α/β treatment. (I) Data are expressed as fold change in titer between IFN-α/β-treated and untreated cells at each temperature ± SD. *, *P* < 0.05, two-tailed Student’s *t* test of log-transformed fold change values. All infections were performed in triplicate, and data are representative of at least two independent experiments. See also Fig. S1.

To test the hypothesis that arboviruses are differentially sensitive to IFN-α/β treatment at different temperatures, we compared growth of wild-type strains from three distinct arbovirus genera, *Alphavirus*, *Flavivirus*, and *Phlebovirus*, in Vero cells treated with IFN-α/β at temperatures between 30 and 39°C with growth in temperature-matched untreated cells. Because Vero cells lack the IFN-α/β genes, they circumvent the potential confounding variable of temperature-dependent differences in IFN-α/β induction upon viral infection (15, 16). The arbovirus strains chosen, CHIKV La Reunion (CHIKV-LR), Sindbis virus TR339 (SINV-TR339), Venezuelan equine encephalitis virus ZPC738 (VEEV-ZPC738), eastern equine encephalitis virus FL93939 (EEEV-FL93), dengue virus 2 16681 (DENV2-16681), yellow fever virus Angola (YFV-Angola), and Rift Valley fever virus ZH501 (RVFV-ZH501), are wild-type viruses that have undergone no or minimal passage *in vitro*, to limit potential selection for optimal replication under typical laboratory conditions, such as growth at 37°C. In the absence of IFN-α/β pretreatment in Vero cells, alphavirus growth was greatest at 37°C, the normal mammalian core body temperature, by 24 h postinfection (hpi) compared to growth at 30 or 39°C, which was comparable to that at 37°C or slightly attenuated (Fig. 1A; see also Fig. S1A in the supplemental material). This was also true in baby hamster kidney (BHK) cells (Fig. S1B). Thus, in the absence of IFN-α/β, departure from 37°C conferred no advantage on alphavirus replication. Conversely, in cells pretreated with IFN-α/β, incubation at 30°C nearly uniformly conferred significant advantage on virus growth compared to incubation at 37°C, while incubation at 39°C, representing a febrile-range temperature, significantly reduced SINV and CHIKV growth compared to that at 37°C (Fig. 1B). Accordingly, the replication ability of three alphaviruses, EEEV, CHIKV, and SINV, in IFN-α/β-treated cells was found to be significantly correlated with increasing IFN-α/β treatment temperature (Fig. 1C). These results indicate that IFN-α/β efficacy was lowest at 30°C and increased with rising temperature. No such correlation was observed with VEEV, which is much more IFN-α/β resistant than the other alphaviruses and was minimally inhibited in Vero cells at the IFN-α/β concentration used (36, 37), further supporting the idea that decreased inhibition at 30°C is due to effects on IFN-α/β activity.

10.1128/mBio.00535-18.1 FIG S1  Efficacy of type I IFN against arboviruses is reduced at subnormal temperatures. Related to Fig. 1. (A and B) Unprimed Vero (A) or baby hamster kidney (BHK) (B) cells were infected in triplicate with the indicated alphaviruses at an MOI of 0.1 and incubated at 30, 37, or 39°C. At the indicated times postinfection, supernatants were harvested and viral titer was determined by plaque assay on BHK cells at 37°C. (C) Osteoblasts cultured from adult CD1 mice were treated with 1,000 IU/ml IFN-α/β at 30 or 37°C overnight and then infected at an MOI of 0.1 with the indicated alphaviruses. Twenty-four hours postinfection, supernatants were assessed for viral titer by plaque assay at 37°C. Data are expressed as fold change in titer between IFN-α/β-treated and untreated cells at each temperature. *, *P* < 0.001 using multiple two-tailed *t* tests with Holm-Sidak correction of log-transformed fold change values. (D) MEFs were treated overnight with 100 IU/ml IFN-α/β at 30, 37, or 39°C and infected with YFV-Angola at an MOI of 0.1. Forty-eight hours postinfection, supernatants were assayed for viral titer by focus-forming assay on Vero cells at 37°C. Data are presented as fold change in titer between IFN-α/β-treated and untreated cells at each temperature. *, *P* < 0.0001, one-way analysis of variance with Tukey’s multiple-comparison test of log-transformed fold change values. Download FIG S1, PDF file, 0.4 MB. Copyright © 2018 Lane et al. 2018 Lane et al. This content is distributed under the terms of the Creative Commons Attribution 4.0 International license.

In addition, the 50% effective concentration (EC_50_) of IFN-α/β against SINV, CHIKV, EEEV, and VEEV on Vero cells was significantly correlated with incubation temperature, with substantially lower concentrations of IFN-α/β effectively inhibiting these viruses as temperature increased (Fig. 1D and E). Finally, analogous temperature-dependent IFN-α/β sensitivity was observed in experiments with YFV and DENV as well as RVFV, demonstrating that this effect is relevant to multiple families of arboviruses capable of replicating across a broad temperature spectrum (Fig. 1F to I). Similar results were obtained in NIH/3T3 Tet-Off murine embryonic fibroblasts (MEFs) and primary murine osteoblasts, cell types representative of *in vivo* targets common to many of these viruses (Fig. S1B and C). Collectively, these experiments suggest that the antiviral activities of IFN-α/β are most effective versus a variety of arboviruses at temperatures in the febrile range and much less effective at subnormal temperatures in mammalian cells.

### ISG protein and mRNA levels are reduced at subnormal temperatures.

**FIG 2:**
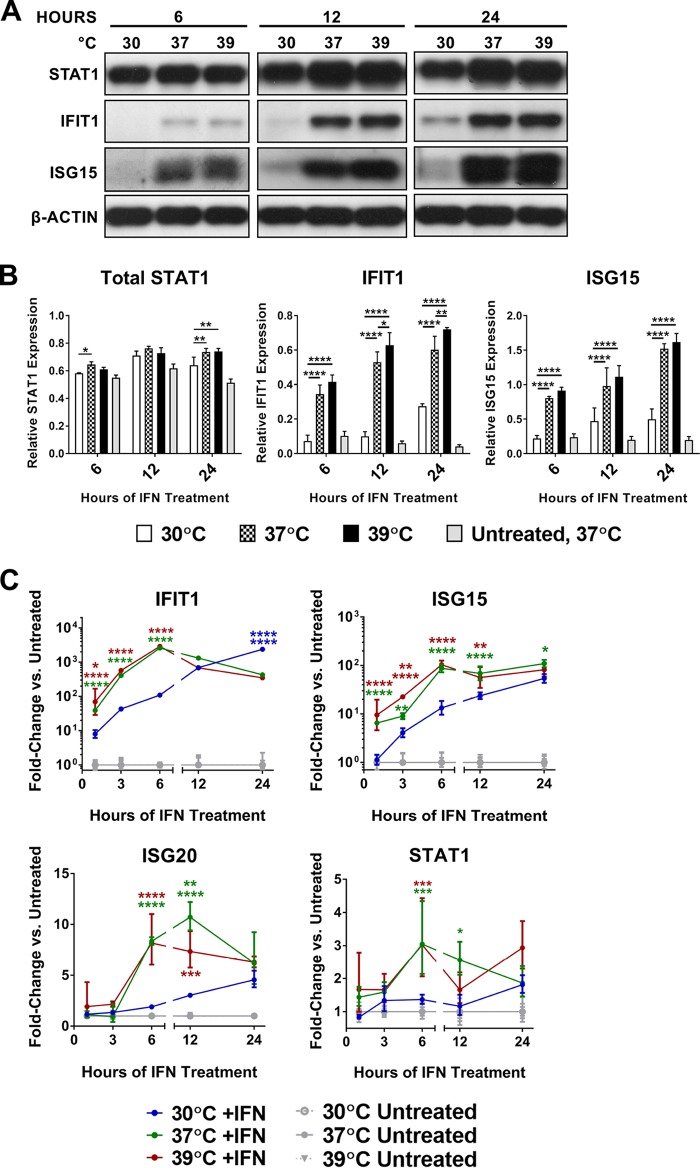
At subnormal temperatures, ISG protein and mRNA levels are reduced. For all panels, Vero cells were treated with 1,000 IU/ml IFN-α/β at 30, 37, or 39°C for 0 to 24 h. (A and B) Immunoblotting of ISG protein levels (A) and corresponding densitometry quantification with ISG bands normalized to β-actin (B), presented as mean ratio of ISG to actin ± SD. (C) qRT-PCR was performed on total cellular RNA with specific primers for the indicated ISGs. Data are presented as fold change in 18S rRNA-normalized *C*_*T*_ values between IFN-α/β-treated and untreated cells at each temperature ± SD. Significance between groups is indicated with color-coordinated asterisks. For example, red and green asterisks indicate significance of 39 and 37°C results, respectively, over 30°C. Two sets of same-colored asterisks stacked over a single data point indicate significance of the results at that temperature over both others at that time point. Statistics: *, *P* < 0.05; **, *P* < 0.01; ***, *P* < 0.001; ****, *P* < 0.0001; ns, not significant by two-way analysis of variance with Tukey’s multiple-comparison test. All experiments were done with triplicate samples, and data are representative of at least two independent experiments. See also Fig. S2.

We hypothesized that the effect of temperature variation on IFN-α/β antiviral efficacy was most likely to result from differential expression levels of antiviral IFN-stimulated genes (ISGs). To test this, we treated cells with IFN-α/β at 30, 37, or 39°C for the time course indicated and performed Western blot analyses and reverse transcription-quantitative PCR (qRT-PCR) for protein and mRNA levels of several ISGs. STAT1, IFIT1, and ISG15 proteins were induced more slowly and to a lower peak expression level at 30°C than at 37°C, and their expression was, in several cases, significantly increased at 39°C versus 37°C (Fig. 2A and B and S2A to C). ISG mRNA expression patterns paralleled protein production at early time points, suggesting that temperature-dependent differences in ISG transcription efficiency contribute to differences in corresponding protein levels (Fig. 2C and S2D and E). However, ISG mRNA induced at 30°C did equal or surpass the levels induced at the higher temperatures at later times post-IFN-α/β treatment, whereas protein levels did not. These data may suggest that both ISG transcription and subsequent translation are independently affected by cell temperature variation and that attenuation of both processes at 30°C contributes to the decreased magnitude of ISG production in response to IFN-α/β treatment.

10.1128/mBio.00535-18.2 FIG S2  At subnormal temperatures, ISG protein and mRNA levels are reduced. Related to Fig. 2. (A to C) Primary CD1 osteoblasts (A), MEFs (B), and HeLa cells (C) were treated with 100 IU/ml IFN-α/β at 30, 37, or 39°C for 6 to 24 h. Lysates were analyzed for ISG protein production by immunoblotting. Graphs display densitometry analysis of ISG bands at each temperature normalized to β-actin. (D and E) The procedure from panels A to C was repeated, and total cellular RNA was probed for ISG mRNA content by qRT-PCR. Data are presented as log_10_ fold change (D) or fold change (E) between 18S rRNA-normalized *C*_*T*_ values for IFN-α/β-treated and untreated cells at each temperature. Statistics: *, *P* < 0.05; **, *P* < 0.01; ***, *P* < 0.001; ****, *P* < 0.0001; ns, not significant by two-way analysis of variance with Tukey’s multiple-comparison test. Download FIG S2, PDF file, 2.5 MB. Copyright © 2018 Lane et al. 2018 Lane et al. This content is distributed under the terms of the Creative Commons Attribution 4.0 International license.

To support this idea, we performed qRT-PCR for the gamma actin intron 3, as a proxy for basal transcriptional rate (37, 38), in cells incubated at different temperatures. We observed an approximately 2-fold decrease in intron transcript levels in cells incubated at 30°C compared to 37°C; however, raising the temperature to 39°C did not appear to influence basal transcription (Fig. S3A). To test whether basal translation rate was also affected by temperature variation separately from effects on basal transcription, we transfected MEF cells with *in vitro*-transcribed RNAs expressing firefly luciferase (fLuc) and measured fLuc activity after 1 h of incubation at 30, 37, and 39°C. At 30°C, fLuc reporter protein levels were slightly lower than those at 37°C, and fLuc activity was slightly increased at 39°C over that at 37°C (Fig. S3B). Beyond 1 h, fLuc activity at 30°C met and surpassed the higher temperatures and continued to rise through 6 h posttransfection, while activity at the higher temperatures peaked and waned, indicating a defect in reporter RNA and/or fLuc protein degradation at 30°C. We confirmed this result in MEF cells lacking *Ifnar1* to rule out differential IFN-α/β responses to the reporter RNA at different temperatures accounting for this result (Fig. S3B). To verify that basal translation rates vary with temperature in a native setting, we performed a [^35^S]methionine-cysteine incorporation pulse-chase experiment in MEFs incubated at 30, 37, and 39°C. Equal volumes of total protein lysates were quantified by SDS-PAGE followed by autoradiography and densitometry analysis of all visible protein bands. In agreement with the fLuc reporter results, we observed an approximately 2-fold reduction in new protein synthesis at 30°C compared to that at the higher temperatures after 1 and 12 h of incubation (Fig. S3C). These results indicate that both basal transcription and translation are affected by cellular temperature and likely contribute to the effect of temperature variation on ISG expression.

10.1128/mBio.00535-18.3 FIG S3  Temperature variation affects baseline transcription and translation rates. (A) Total cellular RNA from MEFs incubated at different temperatures was assayed for gamma actin intron 3 using qRT-PCR. Data are presented as fold change in 18S rRNA-normalized *C*_*T*_ values versus 37°C. (B) Wild-type or *Ifnar1*^*−/−*^ MEF cells were transfected with 5 µg of *in vitro*-transcribed firefly luciferase-expressing reporter RNA and divided among 30, 37, and 39°C temperature conditions for 1, 3, or 6 h. Luciferase translation efficiency was quantified by luciferase activity assay, and RLU values were normalized to total protein content determined by BCA assay. (C) MEF cells were incubated at 30, 37, or 39°C for 1 or 12 h, and new protein production was marked by ^35^S-labeled cysteine and methionine incorporation. Lysates were separated using SDS-PAGE, and total protein was quantified using autoradiography followed by densitometry analysis of all visible bands in each lane. Graph: quantification of data from panel C. Data are presented as fold change versus mean protein content at 37°C at each time point. Download FIG S3, PDF file, 0.4 MB. Copyright © 2018 Lane et al. 2018 Lane et al. This content is distributed under the terms of the Creative Commons Attribution 4.0 International license.

### Suppression of type I IFN signaling pathways is not associated with early temperature effects.

**FIG 3:**
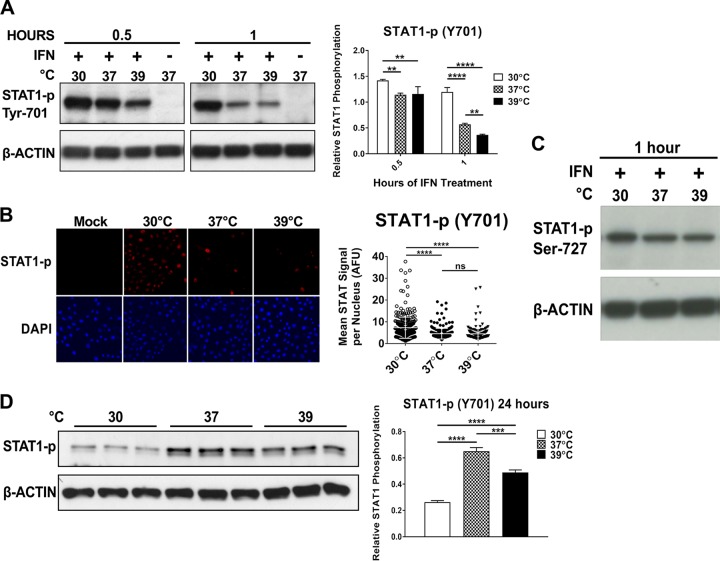
Suppression of type I IFN signaling pathways is not associated with early temperature effects. (A, C, and D) Immunoblot assay of STAT1 phosphorylated at Tyr-701 (A and D) or Ser-727 (C) from Vero cells treated with 1,000 IU/ml IFN-α/β at 30, 37, or 39°C and corresponding densitometry quantification with STAT1-p bands normalized to β-actin. Data are presented as mean STAT1-p/actin ratio ± SD. (B) Vero cells treated with 1,000 IU/ml IFN-α/β for 30 min at 30, 37, or 39°C were subjected to immunocytochemistry staining for STAT1-p (Y701). Confocal imaging shows phosphorylated STAT1 nuclear translocation. Graph displays average nuclear STAT1-p signal intensity per imaged nuclear area at each temperature. At least 199 cells/nucleus were analyzed in each temperature group. Statistics for panel A: **, *P* < 0.01; ****, *P* < 0.0001, two-way analysis of variance with Tukey’s multiple-comparison test. Statistics for panels B and D: ***, *P* < 0.001; ****, *P* < 0.0001; ns, not significant by one-way analysis of variance with Tukey’s multiple-comparison test. All experiments were done with triplicate samples, and data are representative of at least two independent experiments. See also Fig. S4.

In addition to the effects of temperature variation on global cellular transcription rates, we hypothesized that effects on one or more steps in the IFN-α/β signaling cascade may contribute to the temperature sensitivity of ISG transcription. IFN-α/β stimulates gene expression via activation of the JAK-STAT pathway, dependent upon the receptor tyrosine kinase activity of the IFN-α receptor complex (IFNAR1/2) and subsequent phosphorylation of primarily STAT1 and STAT2. It has been published previously that IFN-IFNAR binding characteristics do not vary significantly in the range of 30 to 37°C (39), so we decided first to examine the effect of temperature variation on the phosphorylation of STAT1 at Tyr-701. This step is mediated by the kinase Jak1 at the receptor complex and is required for assembly of the transcription factor complex ISGF3, which stimulates ISG transcription in the nucleus (reviewed in reference 40). Cells treated with IFN-α/β at 30, 37, or 39°C for 30 min showed similar levels of phosphorylated STAT1 (STAT1-p Tyr-701) by immunoblot analysis (Fig. 3A and S4A and B). By 60 min of IFN-α/β treatment, STAT1-p levels remained elevated in the 30°C samples but decreased at the higher temperatures (Fig. 3A and S4B). Because phosphorylated STAT1 is required for ISG transcription, these findings did not fit with the attenuation of ISG transcription that we observed at 30°C (Fig. 2C and S2D and E). We reasoned that despite efficient phosphorylation of STAT1 at 30°C, its nuclear translocation might be negatively affected and result in less activated STAT1 in the nucleus available for ISG transcription. To test this, we examined the efficiency of STAT1-p Tyr-701 migration into the nucleus at different temperatures using confocal microscopy. In agreement with total STAT1-p levels, but in contrast to the observed effect on ISG transcription, IFN-α/β treatment of cells at 30°C resulted in significantly higher average content of STAT1-p per nucleus than at 37°C and 39°C by 30 min of IFN-α/β stimulation (Fig. 3B and S4C). Furthermore, we noted similar levels of STAT1 phosphorylated at Ser-727, a modification required for maximal transcriptional activity of STAT1 that occurs in the nucleus, at all temperatures at 1 h post-IFN-α/β treatment (Fig. 3C and S4D). Interestingly, late after IFN-α/β treatment, levels of STAT1-p Tyr-701 at 37 and 39°C were significantly increased over those at 30°C, indicating a potential mechanism for greater sustenance of the response at higher temperatures (Fig. 3D). This finding could perhaps be a consequence, in part, of the increased level of total STAT1 present at the higher temperatures compared to that at 30°C at this time point (Fig. 2A and B).

10.1128/mBio.00535-18.4 FIG S4  Suppression of type I IFN signaling pathways is not associated with early temperature effects. Related to Fig. 3. (A, B, and D) MEF and HeLa cells were treated with 100 IU/ml IFN-α/β for 30 or 60 min at 30, 37, or 39°C, and lysates were probed for STAT1 phosphorylated at Tyr-701 (A and B) or Ser-727 (D) by immunoblotting. Graphs display densitometry analysis of STAT1-p bands normalized to β-actin at each temperature. Statistics for panels A and B: *, *P* < 0.05; **, *P* < 0.01; ***, *P* < 0.001; ****, *P* < 0.0001; ns, not significant by two-way analysis of variance with Tukey’s multiple-comparison test. (C) HeLa cells treated with 1,000 IU/ml IFN-α/β for 30 min at 30, 37, or 39°C were subjected to immunocytochemistry staining for STAT1-p (Y701). Confocal imaging shows phosphorylated STAT1 nuclear translocation. Graph displays average nuclear STAT1-p signal intensity per imaged nuclear area at each temperature. ****, *P* < 0.0001, one-way analysis of variance with Tukey’s multiple-comparison test. Download FIG S4, PDF file, 0.7 MB. Copyright © 2018 Lane et al. 2018 Lane et al. This content is distributed under the terms of the Creative Commons Attribution 4.0 International license.

### Transcription of IFN response pathway genes is highly temperature dependent.

Because the IFN-α/β signaling pathway upstream of ISG transcription was not attenuated by subnormal cellular temperature, we decided to focus more closely on the effect of temperature variation on ISG transcription. Specifically, we examined whether ISG mRNA induction was unique in its temperature sensitivity compared to other inducible and constitutively expressed genes or whether this phenotype might be solely attributable to a decrease in global transcription rates. We performed direct mRNA quantification analysis via the NanoString platform on total RNA derived from MEF cells treated at 30, 37, or 39°C with IFN-α/β or lipopolysaccharide (LPS) for 3 to 12 h. NanoString is a high-throughput mRNA quantification method that utilizes fluorescently bar-coded hybridization probes to simultaneously detect and count hundreds of distinct mRNA species in a given sample. Our multiplexed gene target panel included a variety of inducible genes from each stimulus, as well as signaling intermediates, feedback and regulatory genes, and cell homeostatic components such as transcription and translation factors (see Table S2 for a complete list of target genes). Most constitutive genes were not appreciably affected by changing temperature or the addition of either stimulus (data not shown), including genes selected for normalization (Fig. S5A). Expression of known temperature-responsive genes, including the cold-inducible CIRP and RBM3 and the heat-inducible HSP70, displayed the expected temperature-dependent patterns, but expression was not influenced by IFN-α/β or LPS treatment (Fig. S5B). Gamma actin intron 3 levels also followed the temperature-sensitive trend that we observed in our qRT-PCR assays (Fig. S5C). These results combine to validate the NanoString platform as a reliable method for quantitating the effect of temperature variation on gene expression.

**FIG 4:**
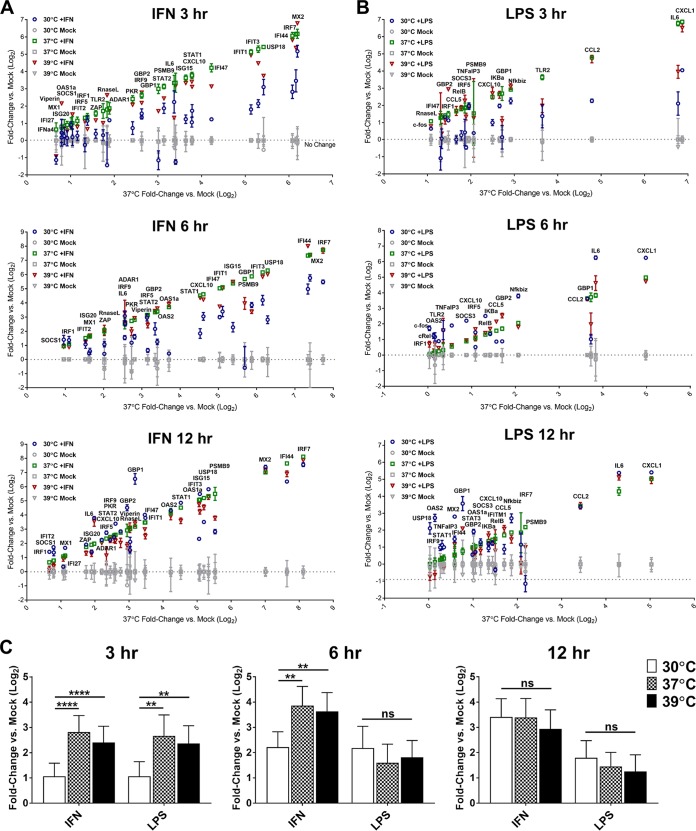
Transcription of IFN response pathway genes is highly temperature dependent. NIH/3T3 MEF cells were treated with 100 IU IFN-α/β or 250 ng/ml LPS, or were left untreated, at 30, 37, or 39°C for the time course indicated in duplicate samples. At each time point, total cellular RNA was harvested and subjected to direct mRNA quantification of 125 gene targets (see Table S2) using the NanoString platform. Raw mRNA counts in each sample were subjected to background subtraction followed by normalization to the geometric mean of five normalization genes (β-actin, glyceraldehyde-3-phosphate dehydrogenase [GAPDH], β-tubulin, POLR2A, and eIF3a) in the same sample. (A and B) Genes upregulated at least 2-fold in treated cells versus mock at any temperature are shown as log_2_ mean fold change of duplicate samples versus mock. Genes are ordered along the *x* axis by log_2_ fold change in expression at 37°C. (C) Bar graphs show geometric mean ± SD of ≥2-fold-upregulated genes at each temperature at each time point. Statistics: **, *P* < 0.01; ****, *P* < 0.0001; ns, not significant by Mann-Whitney rank test. See also Fig. S5.

10.1128/mBio.00535-18.5 FIG S5  NanoString analysis detects temperature-sensitive gene transcription. Related to Fig. 4. MEF cells were treated in with 100 IU IFN-α/β or 250 ng/ml LPS, or were left untreated, at 30, 37, or 39°C for the time course indicated in duplicate samples. At each time point, total cellular RNA was harvested and subjected to direct mRNA quantification of 125 gene targets (see Table S2) using the NanoString platform. Raw mRNA counts in each sample were subjected to background subtraction followed by normalization to the geometric mean of five normalization genes (β-actin, glyceraldehyde-3-phosphate dehydrogenase [GAPDH], β-tubulin, POLR2A, and eIF3a) in the same sample. (A) Background-subtracted mRNA count values of the five genes used for normalization, with IFN-α/β- or LPS-stimulated and unstimulated samples pooled at each temperature. (B) Normalized expression profile of three known temperature-responsive genes, with IFN-α/β- or LPS-stimulated and unstimulated samples pooled at each temperature. (C) Gamma actin intron 3 expression. (D) Interleukin-6 (IL-6) expression in LPS-treated MEFs in an independent experiment, quantified by qRT-PCR. (E) Verification of expression of other LPS-responsive genes in MEFs at 3 h post-LPS treatment in an independent experiment, quantified by qRT-PCR. Download FIG S5, PDF file, 0.7 MB. Copyright © 2018 Lane et al. 2018 Lane et al. This content is distributed under the terms of the Creative Commons Attribution 4.0 International license.

Figure 4A shows the fold induction of IFN-α/β-responsive genes at 30, 37, and 39°C versus temperature-matched mock-treated samples at 3, 6, and 12 h post-IFN-α/β treatment, respectively. Figure 4B shows analogous results for LPS-treated cells. Only genes upregulated at least 2-fold over mock at any temperature are shown and included in subsequent analyses. After 3 h of stimulation with IFN-α/β or LPS, IFN-α/β-induced and LPS-induced genes displayed similar patterns of temperature-sensitive expression in general, with optimal induction at 37°C or 39°C and dramatically reduced induction at 30°C (Fig. 4A and B). Upon closer examination, however, differences became apparent. Most ISGs in our panel displayed decreased expression at 30°C compared to 37°C far below the 2-fold decrease in basal transcriptional rate that we observed (Fig. S3A and S5C), indicating that the temperature sensitivity of ISG transcription may be attributable to additional mechanisms. Although this was also true of some LPS-inducible genes, there were several examples (IKBA, NFKBIZ, and TNFAIP3) upon which temperature variation seemed to have little or no effect (Fig. 4B). These results contrasted with the reliable temperature sensitivity of all ISGs in the panel. In addition, induction kinetics varied between ISGs and LPS-induced genes throughout the rest of the time course. For LPS-induced genes, apart from known ISGs also induced by LPS (GBP1 and GBP2), expression in the 30°C group matched or exceeded expression at the higher temperatures by just 6 h of LPS treatment (Fig. 4B and C). At 30°C in IFN-α/β-treated cells, in contrast, ISG induction remained significantly lower than at higher temperatures until 12 h of IFN-α/β treatment (Fig. 4C). These differences in gene induction by LPS and IFN-α/β at different temperatures could indicate gene- or pathway-specific responses to changing cellular temperature beyond simple changes to basal transcriptional rates.

### Reduced temperature also affects type I IFN induction.

**FIG 5:**
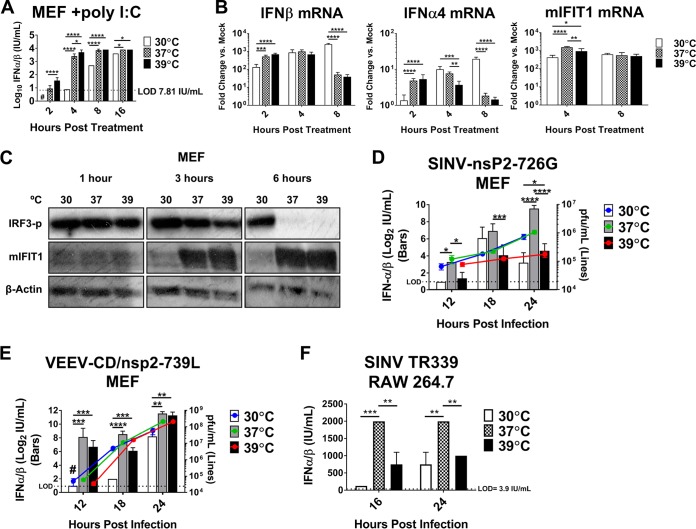
Reduced temperature also affects IFN-α/β induction. (A to C) MEFs were transfected with 50 µg poly(I:C) per million cells using electroporation. Pooled transfections were then divided among 30, 37, and 39°C temperature conditions. (A) Supernatants were assayed for biologically active IFN-α/β as described in Materials and Methods. Data are presented as log_10_ mean IU per milliliter ± SD. (B) qRT-PCR was used to quantify IFN-α/β and IFIT1 mRNA induction. Data are presented as mean fold change in 18S rRNA-normalized *C*_*T*_ values in poly(I:C)-treated versus untreated cells at each temperature ± SD. (C) Immunoblot assay of IRF3 phosphorylated at Ser-396 and IFIT1 from poly(I:C)-treated MEF lysates. (D to F) Supernatants from virus-infected MEFs or RAW 264.7 cells were assayed for IFN-α/β as in panel A. IFN-α/β induction data are presented on the left *y* axis as log_2_ mean IU per milliliter ± SD. Statistics: *, *P* < 0.05; **, *P* < 0.01; ***, *P* < 0.001; ****, *P* < 0.0001; ns, not significant by two-way ANOVA with Tukey’s multiple-comparison test of log-transformed IU/milliliter values. Viral titer data for panels D and E were determined by plaque assay on BHK cells at 37°C from the same supernatants assayed for IFN-α/β activity and are presented along the right *y* axis of those panels, ± SD. #, result was below the limit of detection (LOD) of the assay. All experiments were done in duplicate or triplicate, and results shown are representative of at least two independent experiments.

Thus far, our studies have focused on the effect of temperature variation on the signaling/effector phase of the IFN-α/β response. However, as the effector phase is preceded by the IFN-α/β inductive phase in response to infection *in vivo*, it is relevant to examine the effect of temperature in this context as well, as IFN-α/β efficacy *in vivo* depends concurrently on the two phases. It has been published previously that IFN-α/β induction by rhinovirus is diminished at the subnormal temperatures of airway epithelial cells, which could contribute to enhanced rhinovirus fitness in the upper airways (15). To avoid temperature-dependent differences in viral replication that could influence IFN-α/β production, we electroporated poly(I:C) into MEFs and then incubated the cells at 30, 37, or 39°C. We observed the earliest detectable IFN-α/β activity in cell supernatants from the 39°C samples, and IFN-α/β activity remained highest at this temperature throughout the time course (Fig. 5A). IFN-α/β induction at 37°C was slightly but significantly reduced compared to that at 39°C at early time points but still far exceeded levels of IFN-α/β produced at 30°C, which did not surpass the limit of detection until 8 h posttransfection (Fig. 5A). We also performed qRT-PCR analysis for *Ifna4*, *Ifnb*, and *Ifit1* mRNAs and found a similar trend in temperature-dependent expression at early time points (Fig. 5B). Interestingly, while transcript levels of the IFN-α/β genes fell dramatically at the higher temperatures by 8 h post-poly(I:C) treatment, they continued to rise at 30°C (Fig. 5B); however, this did not correspond to increased secreted IFN-α/β at this temperature (Fig. 5A), suggesting that translation suppression and/or attenuation of the secretory pathway may also play a role in temperature-dependent IFN-α/β induction.

The IFN-α/β genes are downstream targets of IRF3, which is activated in response to poly(I:C); therefore, we examined IRF3 activation by Western blotting for IRF3 phosphorylated at Ser-396 (IRF3-p). Temperature variation had no impact on levels of IRF3-p initially, but surprisingly, IRF3-p levels were maintained at 30°C through 6 h poststimulation, while all but disappearing at the higher temperatures (Fig. 5C). In the same samples, however, IFIT1, which is directly IRF3 inducible (7), was much more highly expressed at 37 and 39°C than at 30°C (Fig. 5C). These results underline the discrepancy between activation of the IFN-α/β induction pathway and functional IRF3-responsive gene expression and highlight differences in gene transcription and translation as the major mediators of the effects of temperature variation on these pathways.

The above-described experiments served to examine the effect of temperature variation on the cytosolic IFN-α/β induction pathway with equal initial stimulation of the pathway at all temperatures [i.e., an equal dose of the nonreplicating poly(I:C)]. However, this does not accurately represent the context of a viral infection, in which differences in viral replication rates at different temperatures could lead to variation in pathway stimulation and likely compound the effect of temperature on IFN-α/β induction that we observed with poly(I:C). To verify that viral infection at different temperatures also stimulates differential IFN-α/β induction, we infected cells at 30°C for the first hour to allow equal attachment and entry. After washing, infected wells were divided among 30, 37, and 39°C temperature treatments for 12 to 24 h, at which times supernatants were collected and assayed for IFN-α/β activity as described in Materials and Methods. Infections of MEF cells required the use of mutant viruses whose transcription and translation inhibitory mechanisms have been disabled (SINV-nsP2-726G [Fig. 5D] and VEEV-CD/nsP2-739L [Fig. 5E]) (37). However, we also tested a wild-type alphavirus, SINV-TR339 (Fig. 5F), in the murine monocyte/macrophage RAW 264.7 cell line, which, unlike MEFs, can still produce IFN-α/β upon wild-type alphavirus infection. Unlike the results with poly(I:C), we observed the greatest IFN-α/β activity from the 37°C infections, and levels of IFN-α/β produced at 39°C either did not differ from or were significantly lower than those observed from the 37°C treatment (Fig. 5D to F). In agreement with the poly(I:C) results, however, viral infection at 30°C resulted in greatly delayed and stunted IFN-α/β production (Fig. 5D to F). Surprisingly, viral titers did not vary appreciably between temperatures, in general, indicating that differences in pathway stimulation may not have contributed much to differences in IFN-α/β output in this context. Viral growth at any temperature did not seem to be impacted by IFN-α/β induction by 24 hpi, which may be explained by the fact that these viruses are competent for some inhibition of downstream IFN-α/β signaling in MEFs. The exception was SINV-nsP2-726G at 39°C, which did suffer some inhibition compared to the other temperatures, a possible indication of the increased efficacy of IFN-α/β at 39°C apparent against this virus. Importantly, these results demonstrate that IFN-α/β induction is also sensitive to temperature variation and that a viral infection occurring at subnormal temperatures may benefit both from diminished detection and IFN-α/β induction and from reduced ISG upregulation leading to a weakened antiviral state relative to higher temperatures. Combined, these two factors may create an environment in which the virus can replicate uninhibited by the IFN-α/β response long enough to produce IFN-α/β antagonists, which could then combat subsequent IFN-α/β-mediated restriction, an interplay that could be particularly relevant *in vivo*.

### Lower temperatures *in vivo* suppress IFN-α/β responses.

**FIG 6:**
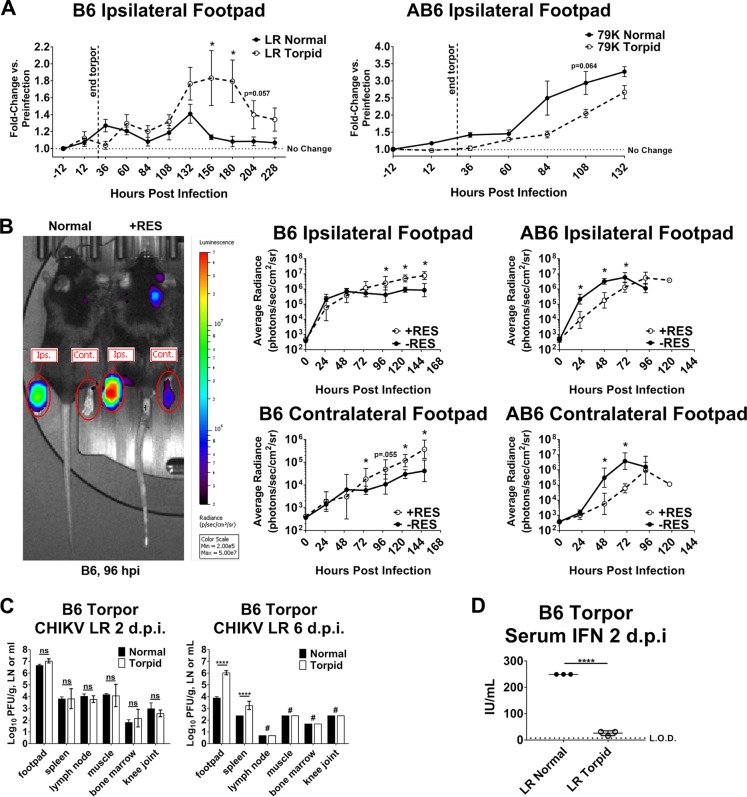
Lower temperature *in vivo* suppresses IFN-α/β responses. (A) Core temperature reduction in 6-week-old C57BL/6 mice and *Ifnar1*^−/−^ AB6 mice was achieved by induction of metabolic torpor. Torpid and normal mice were then infected in the left hind footpad with 1,000 PFU of wild-type CHIKV-LR (B6 mice) or the attenuated variant CHIKV-LR-E279K (AB6 mice). Virus-induced musculoskeletal disease was quantified daily by manual caliper measurement of footpad width and thickness to determine cross-sectional area. Data are presented as fold change in infected footpad cross-sectional area versus preinfection area ± standard error of the mean. (B) Mouse core temperature reduction was induced with intraperitoneal administration of reserpine approximately 3 h prior to infection as in panel A with 1,000 PFU of CHIKV-LR-nluc-TaV, and viral replication was tracked over time using *in vivo* imaging. Data are presented as the geometric mean of average footpad radiance in each group ± geometric SD. Statistics for panels A and B: *, *P* < 0.05, Mann-Whitney rank test at individual time points. (C) Tissues from CHIKV-LR-infected normal and torpid B6 mice were assayed for viral load by plaque assay at day 2 and day 6 postinfection. Data shown are mean viral titers ± SD. ****, *P* < 0.0001; ns, not significant by two-tailed Student’s *t* test of log-transformed titer values. #, titer was below the limit of detection. (D) Serum from CHIKV-LR-infected normal and torpid B6 mice was assayed for biologically active IFN-α/β as described in Materials and Methods. L.O.D., limit of detection. Data are shown as mean IU per milliliter ± SD. ****, *P* < 0.0001 by two-tailed Student’s *t* test. For all experiments, *n* was 3 to 6 mice per group, and data are representative of at least two independent experiments. See also Fig. S6.

To test the possibility that temperature variation might affect the pathogenesis and disease outcomes of a relevant infection *in vivo*, we adapted the C57BL/6 adult murine model of CHIKV infection and musculoskeletal disease (MSD) (41) to include systemic body temperature reduction. One of two strategies was used to achieve core temperature reduction depending on the experimental setup: either induction of metabolic torpor, a prolonged state of inactivity and energy conservation that attenuates heat-generating mechanisms (42), or administration of the small molecule reserpine (RES), which depletes peripheral monoamine neurotransmitters by antagonizing the vesicular monoamine transporter (VMAT), leading to dysregulation of temperature regulatory mechanisms (43, 44). Both methods effectively reduced mouse subcutaneous (scruff) temperature in both wild-type C57BL/6 (B6) and IFNAR1^−/−^ (AB6) mice (Fig. S6). For assessing the effect of reduced temperature on CHIKV-induced MSD, torpid and normal mice were infected in the left rear footpad and cross-sectional area was measured daily to track foot swelling. In wild-type mice, torpor induction resulted in significantly greater MSD between 6 and 8 days postinfection (dpi), whereas in IFNAR1^−/−^ AB6 animals, MSD was slightly reduced by torpor (Fig. 6A). These results suggest that the effect of reduced body temperature on CHIKV-induced disease signs manifests in an IFN-α/β-dependent manner, with low temperature leading to more severe disease only when functional IFN-α/β is present.

10.1128/mBio.00535-18.6 FIG S6  Torpor and reserpine treatment reduce mouse core temperature. Related to Fig. 6. Mice were implanted subcutaneously in the scruff with temperature transponders (BMDS) prior to administration of reserpine (A) or induction of metabolic torpor (B) as described in Materials and Methods. Temperature was monitored regularly throughout the course of all experiments, and graphs represent the typical temperature profiles of reserpine-treated or torpid animals versus normal. Download FIG S6, PDF file, 0.4 MB. Copyright © 2018 Lane et al. 2018 Lane et al. This content is distributed under the terms of the Creative Commons Attribution 4.0 International license.

Next, we used RES treatment to extend the duration of temperature reduction and examine viral growth kinetics using a nanoluciferase (nLuc)-expressing CHIKV (45) and *in vivo* imaging. In B6 mice, viral signal in both the infected (ipsilateral) and contralateral footpads rose similarly over time between RES-treated and normal animals until 3 days postinfection, beyond which the signal in the RES-treated group increased significantly over the signal in the warm group (Fig. 6B). An opposite trend was observed in AB6 mice, with viral signal in RES-treated animals significantly lower than in the warm mice for the entire course of infection, until the mice were about to succumb to the infection (Fig. 6B). In addition, viral titers from dissected tissues were not significantly different between normal and torpid B6 mice at 2 dpi but remained high in the infected footpad and spleen of the torpid mice by 6 dpi, indicating dysfunctional viral clearance in those tissues at reduced temperature (Fig. 6C). Finally, torpid mice failed to induce a robust serum IFN-α/β response at 2 days post-CHIKV infection compared to normal mice, suggesting that impaired IFN-α/β inductive pathways *in vivo* at low temperatures likely contribute to the observed effects on CHIKV infection (Fig. 6D), although kinetic differences in virus replication brought on by the altered temperatures may have contributed to this result.

## DISCUSSION

Historically, studies of the IFN-α/β response have largely relied on genetic knockout strategies to examine the roles of individual mediators and effectors in various infection and disease models. However, there is a growing body of literature focused on intact innate immune mechanisms within the variable physiological contexts in which they operate *in vivo* and how this variability may affect efficacy (15, 16, 19, 20, 23, 46–49). The results presented here indicate that ambient temperature can have broad impacts on the effectiveness of the mammalian IFN-α/β antiviral response. Many human pathogens, such as respiratory viruses, and other pathologies, such as certain malignancies (50), cause disease in tissues regularly exposed to temperatures below the normal core temperature of 37°C and may take advantage of a weakened innate immune environment. In these studies, we examined whether arboviruses, which are replication competent across a wide range of temperatures due to their evolution in both arthropod vectors and mammalian or avian hosts, represented another type of pathogen capable of subverting IFN-α/β responses occurring at subnormal temperatures. *In vitro*, we found that arboviruses representing three distinct viral families had increased fitness at 30°C relative to 37°C but only when cells had been pretreated with IFN-α/β at each temperature. Without IFN-α/β present, lowered temperature conferred no advantage on viral replication. These findings parallel trends observed with human rhinovirus (RV) and influenza virus (15, 16), although the increased fitness of RV at low temperature is attributable to additional mechanisms as well (51). Uniquely, our studies demonstrate that temperature-dependent IFN-α/β efficacy alone is sufficient to alter arbovirus fitness and effectively overcome the small disadvantage posed to these viruses by low temperature in the absence of IFN-α/β. Collectively, these data suggest that the effect of temperature on IFN-α/β efficacy is widely applicable to any IFN-α/β-sensitive infection or pathology that occurs in a reduced-temperature environment.

To examine the effect of temperature on the entirety of the IFN-α/β response, we examined both IFN-α/β gene upregulation and ISG upregulation phases. Mechanistically, it has been shown previously (15, 16) that several ISGs were less robustly induced at the mRNA level when cells were treated with IFN-α/β at temperatures below 37°C, but the mechanism(s) leading to decreased ISG transcription, as well as whether reductions in ISG transcript are functionally relevant to the antiviral state at the protein level, was not addressed. We found that activation of the JAK-STAT pathway resulting in phosphorylation, nuclear translocation, and nuclear phosphorylation of STAT1 to the transcription-promoting state was not attenuated by subnormal temperature. In fact, IFN-α/β signaling at 30°C resulted in more phosphorylated STAT1 present in the nucleus than signaling at 37°C. Thus, differences in IFN-α/β signaling efficiency were not responsible for the observed reduction in ISG transcript induction at subnormal temperature. Importantly, marked temperature-dependent differences in ISG expression were observed at the protein level, indicating that differences in ISG transcription and possibly subsequent translation result in differential functionality of the antiviral state at different temperatures. It is also possible that the activities of individual ISGs may be affected by temperature variation and that this could contribute to differences in their efficacy. If true, this idea may help explain the enhanced antiviral activity of IFN-α/β observed at 39°C versus 37°C, when ISG protein levels were only modestly higher at this temperature. Moreover, enhanced induction of ISGs known to strengthen the IFN-α/β response, such as RIG-I or RNase L, at higher temperatures may also contribute to increased efficacy versus lower temperatures, although this idea would presumably apply also to negative-feedback ISGs, such as SOCS. Further studies will be needed to test this idea for individual ISGs and to determine the relative contributions that these differences may make to immunity against specific pathogens. In sum, we conclude that transcription is the first step in both the IFN-α/β inductive and effector ISG inductive pathways affected by low cellular temperature that lead to attenuation of the antiviral activity of the IFN-α/β response.

Although detailed examination of the transcription factor activities for individual ISGs that account for the effect of temperature was beyond the scope of our current studies, our results comparing baseline transcriptional rate with ISG and LPS-responsive gene transcriptional efficiency did reveal some relevant trends. Transcript levels of the constitutively expressed genes included in our NanoString analysis did not change appreciably upon temperature shift, while initial transcription of genes induced upon IFN-α/β or LPS treatment was highly affected by incubation temperature. We observed an approximately 2-fold reduction in basal transcriptional rate at 30°C versus 37°C by intron qRT-PCR, and this may account for a portion of the reduction in inducible gene expression. However, it does not fully explain the dramatic differences in induction of all ISGs that were highly upregulated, nor does it account for the subset of LPS-responsive genes whose expression was not temperature sensitive. In general, these results point to a model in which inactive genes are much more susceptible to transcription attenuation by low temperature than genes actively being transcribed. Potentially, chromatin remodeling around inducible promoters and initial assembly of transcription machinery represent additional obstacles to inducible genes being “turned on” that are absent from constitutively active genes (52). In addition, variation in the chromatin environments around different promoters, as well as in the transcription factors required for transcription of different genes, would allow for differential temperature sensitivity of inducible genes. Indeed, increased sp1 recruitment to a hypothermia-responsive element upstream of the cold-responsive RNA binding protein (*Cirp*) locus has been identified in the preferential upregulation of this gene at low temperature (53), indicating that temperature shift can impact transcription initiation at the chromatin level. In this view, the reliable temperature sensitivity that we observed with ISGs is consistent with the presumption that IFN-stimulated response element (ISRE)-containing promoters reliant on similar transcription factors and chromatin modifications (54–56) would be subject to the same challenges of transcription initiation at subnormal temperatures, yielding the phenotype of temperature-sensitive ISG transcription. In contrast, the chromatin remodeling events and transcription factors required for LPS-responsive genes can vary considerably (52, 57), allowing for the possibility of different effects of temperature shift on individual genes or subsets of genes. Consistent with this idea, several genes that responded to both LPS and IFN-α/β treatment followed the same temperature-dependent expression patterns regardless of the stimulus. Examination of additional signaling cascades with known repertoires of inducible genes, particularly those that stimulate distinct sets of transcription factors and cofactors to effect transcription of different gene subsets, will be helpful in elucidating gene-specific versus pan-regulatory effects of temperature variation.

The initial lag in ISG transcription at 30°C was largely overcome by 12 h post-IFN-α/β treatment, and in some cases, ISG transcript levels at this temperature continued to increase as they began to stabilize or wane at the higher temperatures. Similarly, LPS-inducible gene transcript levels at 30°C also initially lagged behind those at higher temperatures on average but drew level by just 6 h of LPS treatment, with many genes exhibiting greater expression at 30°C by this time point. Although the mechanisms behind this temporal difference in ISG and LPS-induced gene regulation by temperature are unclear, this observation represents another piece of evidence that the effect of temperature on gene transcription is likely gene and/or pathway specific. That levels of gene transcripts induced at 30°C eventually met or exceeded peak expression at the higher temperatures and remained elevated over a longer period of time could be the result of a generalized increase in mRNA stability at subnormal temperatures (58–60) and/or due to a defect in negative-feedback pathways responsible for curtailing these signaling pathways. Regardless, there did not appear to be a functional consequence of ISG mRNA levels “catching up” at 30°C, as ISG protein levels remained reduced at this temperature compared to 37 and 39°C, even as late as 48 h post-IFN-α/β treatment. It is likely that protein translation was also attenuated separately from transcription, accounting for this difference, as translational slowing is generally considered a hallmark feature of the cellular cold stress response (59, 61). This idea is supported by our *in vitro* RNA translation reporter and protein synthesis radiolabel assays (see Fig. S3 in the supplemental material). Differences in translational efficiency may also explain instances of enhanced ISG protein production at 39°C over 37°C when there was no difference at the transcript level. We conclude that general suppression of translation likely contributes to the suppression of ISG protein production at 30°C independently from effects on transcription. Whether ISG transcripts are specifically sensitive to temperature-induced changes to translation efficiency, or whether these effects manifest with all mRNAs, is still unclear.

We also investigated the effect of temperature variation on IFN-α/β induction efficiency upon stimulation with either poly(I:C) or viral infection. Poly(I:C), a nonreplicating stimulus, was used for examination of IFN-α/β induction with equal pathway stimulation at each temperature. In agreement with previous studies (15), poly(I:C) treatment of cells at subnormal temperatures resulted in delayed and stunted IFN-α/β production compared to 37°C. That study attributed this difference to a modest reduction in the enzymatic activities of the cytosolic sensors RIG-I and MDA-5 at low temperature, as determined in *in vitro* ATPase assays (15). In contrast, we found that that a downstream consequence of RIG-I/MDA-5 stimulation, the phosphorylation of IRF3, was not suppressed at the low temperature, suggesting that any effect on the enzymatic activity of these sensors is functionally inconsequential in this context. Rather, the effect of temperature on this pathway, as in the JAK-STAT pathway, likely manifests first at the level of transcription of responsive genes. We observed delayed transcription of *IfnB*, *Ifna4*, and *Ifit1* genes, each primarily dependent on IRF3 in fibroblasts (7), at 30°C versus the higher temperatures at early time points, but levels surpassed those at the higher temperatures quickly thereafter. However, IFN-α/β protein release was drastically slowed and reduced at 30°C, both initially and well after mRNA levels had recovered, as was IFIT1 protein expression, indicating that efficiency of translation and/or secretory mechanisms also contributed to the reduction in IFN-α/β secretion at the lower temperature. Further studies are required to determine whether translation or secretory suppression by low temperature represents a universal effect or whether different mRNAs are affected to various degrees. Poly(I:C) treatment at 39°C resulted in the highest induction of IFN-α/β and IFIT1 proteins, indicating that enhanced IFN-α/β production may be an evolutionary aspect of the febrile response *in vivo*. As IFN-α/β is itself a pyrogenic cytokine, this relationship could be evidence of a feed-forward loop in which innate immune responses and fever coregulate to mount a more rapid response to infection.

IFN-α/β induction was similarly temperature dependent upon alphavirus infection, with the cells infected at 30°C resulting in delayed and diminished IFN-α/β production compared to 37°C. Interestingly, we did not observe the same enhancement of peak IFN-α/β production at 39°C compared to 37°C that we did with poly(I:C) treatment. It is reasonable to hypothesize that an enhanced downstream antiviral state at a febrile temperature could lead to more efficient control of the infection, possibly with lower peak IFN-α/β induction. Indeed, we did observe greater viral control of SINV-nsP2-726G at 39°C than at 37 or 30°C in our MEF cultures, even with IFN-α/β levels well below those at 37°C. In contrast, at 30°C, IFN-α/β induction and subsequent effector phase are both delayed and attenuated. Because alphaviruses have evolved to grow efficiently at this temperature (and colder) in arthropods, virus replication largely resists the low-temperature environment, and initial replication proceeds largely uninhibited by IFN-α/β. During this window, the virus can produce sufficient levels of IFN-α/β antagonists before a strong antiviral response can be mounted. IFN-α/β antagonism by most alphaviruses depends on the activities of nonstructural protein 2 (nsP2) or capsid proteins (37, 62, 63), and the viruses can also interfere with JAK-STAT signaling (36, 64, 65).

In a murine model of CHIKV infection, we demonstrated that hypothermia can exacerbate virus replication and associated musculoskeletal disease, but only in the presence of a functional IFN-α/β response. In mice lacking IFNAR1, lowered body temperatures reduced both viral replication and disease signs, indicating that the effect of the reduced temperatures on the virus was acting through its effects on IFN-α/β, and serum IFN-α/β levels were much reduced in cold mice versus their warm counterparts. Surprisingly, differences in MSD in wild-type mice manifested several days after hypothermic mice had recovered to a normal body temperature, indicating that, as predicted from our cell culture models, influences of reduced temperature on viral infection occur at an early stage of the virus-host interaction, in the window before IFN-α/β can control the infection. As MSD in CHIKV infection is a complicated process driven by both viral and host factors (41), it is tempting to speculate that subnormal temperatures early during infection allowed the virus to establish a more severe infection in the face of a weakened IFN-α/β response, which then attracted increased immune cell infiltrates and induced greater inflammation at the site of infection. We did observe delayed viral clearance in the infected footpad and the draining lymph node of the hypothermic mice compared to the normal mice at 6 dpi, which could help drive the exacerbated MSD at later time points. Together, these results demonstrate that CHIKV or other arbovirus pathogenesis and disease can be altered by variation in tissue temperature, with subnormal temperatures benefitting, and supranormal temperatures impairing, virus infection via effects on the IFN-α/β response. This suggests that local or systemic warming could represent a viable therapeutic for infection with CHIKV or other arthritogenic arboviruses if applied early after infection. Finally, it is also worth noting that early studies of temperature and interferon responses focused on the establishment of persistent infection and its relationship to temperature-dependent interferon induction *in vitro* (66–70). It will be of interest to determine if temperature effects on the innate immune system influence the establishment of arboviral persistence in sites such as the testes (Zika virus [71–73]) and peripheral joints (CHIKV [74]) that may differ from core temperatures.

## MATERIALS AND METHODS

### Cell lines.

Baby hamster kidney (BHK-21) cells (ATCC; RRID CVCL_1915) and L929 murine fibrosarcoma cells (RRID CVCL_0462) were maintained in RPMI 1640 supplemented with 10% donor calf serum (DCS) and 10% tryptose phosphate broth (TPB). RAW 264.7 murine monocyte/macrophage (RRID CVCL_0493), African green monkey kidney (Vero; RRID CVCL_0059), and human cervical carcinoma (HeLa; ATCC; RRID CVCL_0058) cells were maintained in Dulbecco’s modified Eagle’s medium (DMEM) supplemented with 10% fetal calf serum (FCS). MEF/3T3 Tet-Off murine embryonic fibroblasts (simply called MEFs throughout; Clontech; CVCL_KS91) and immortalized primary MEFs from *Ifnar1*^*−/−*^ animals (MEF isolation and immortalization are described in reference 75) were maintained in DMEM containing 10% FCS, 100 mM HEPES buffer, 0.075% sodium bicarbonate, and 0.05 mg/ml G418 sulfate (Mediatech). All media also contained 100 U/ml penicillin G sodium and 100 µg/ml streptomycin sulfate. Except for instances of experimental temperature variation, all cells were grown at 37°C in a humidified chamber with 5% CO_2_.

### Primary cell cultures.

Primary murine osteoblast cultures were generated from 3- to 5-day-old suckling CD1 mice and maintained as described previously (76). Briefly, dissected calvaria were triply digested with collagenase P and cultured for 5 days in Minimum Essential Medium Alpha (AMEM) containing 15% FCS at 37°C and 5% CO_2_ in a humidified chamber to allow osteoblast outgrowth. Osteoblasts were detached by trypsinization, strained to remove bone fragments, seeded into 150-mm culture dishes, and allowed to expand under the same growth conditions as described above. Upon reaching confluence, the osteoblasts were trypsinized and seeded into multiwell plates for experimentation.

### Virus stocks.

cDNA clones of SINV-TR339 (77), CHIKV-LR (78), EEEV-FL93-939 (62), and VEEV-ZPC738 (79) have been described elsewhere. Mutant viruses SINV-nsP2-726G (80), VEEV-CD-nsp2-739L (37), and CHIKV-E2-79K (81) were generated by site-directed mutagenesis with appropriate overlapping primers and the QuikChange kit (Agilent) according to the manufacturer’s instructions. The nanoluciferase-expressing CHIKV-LR-nluc-TaV and CHIKV-LR-E2-79K-nluc-TaV viruses are described in reference 45. To generate virus stocks, infectious, capped RNA was generated by *in vitro* transcription from linearized clones (mMessage mMachine; Ambion) and electroporated into BHK-21 cells using a GenePulser II (Bio-Rad) as previously described (82). Virus-containing supernatants were collected after 24 h, and titers were determined on BHK-21 cells using a standard plaque assay. The DENV2 strain 16681 was a gift from Jared Evans. Viral stocks were generated from a single passage on C6/36 mosquito cells, and titers were determined by focus-forming assay on human hepatocarcinoma Huh7 cells using antiflavivirus D1-4G2-4-15 antibody (ATCC). The YFV-Angola strain was a gift from Alan Barrett, and stocks were made and titers were determined as described previously (83). Experiments involving RVFV strain ZH501 (84) were conducted in the laboratory of Amy Hartman. Propagation and titration of this virus are described in reference 85.

### Animals.

All experiments involving animals were carried out under approval of the Institutional Animal Care and Use Committee of the University of Pittsburgh (protocol 15096749) and in accordance with the recommendations found in the *Guide for the Care and Use of Laboratory Animals* (92). Five- to 6-week-old adult male and female C57BL/6 mice were purchased from Jackson Laboratories (RRID IMSR_JAX:000664) and 5- to 7-week-old adult male and female *Ifnar1*^*−/−*^ mice on the C57BL/6 background were bred in-house. Mice were housed socially (maximum 5/cage for females and 4/cage for males) in specific-pathogen-free (SPF) microisolator cages in our animal biosafety level 3 (ABSL3) facility. Animals were kept on a 12-h light/12-h dark cycle with access to food and water *ad libitum* except in instances of experimental induction of metabolic torpor (detailed below). All mice weighed between 15 and 25 g and were drug and test naive prior to use in these studies. Prior to and upon initiation of experiments, all mice were weighed and checked for disease signs (lethargy, ruffled fur, and hunching) daily to ensure maximal animal welfare in accordance with approved IACUC procedures. Animals of both sexes were divided randomly into experimental groups, such that experiments reflect pooled data from both sexes.

### Antibodies and other reagents.

The following antibodies were used for Western blotting and/or immunostaining: mouse anti-β-actin (BA3R; Invitrogen; RRID AB_10979409), rabbit anti-STAT1 (M-22; Santa Cruz; RRID AB_632434), rabbit anti-ISG15 (H-150; Santa Cruz; RRID AB_2126309), rabbit anti-IFIT1 (Invitrogen catalog no. PA5-27907 for human/nonhuman primate samples; RRID AB_2545383), rabbit anti-IFIT1 (Invitrogen catalog no. PA3-846 for murine samples; RRID AB_1958734), rabbit anti-phospho-STAT1 (Tyr-701) (catalog no. 9171; Cell Signaling Technology, Inc.; used for Western blotting assays; RRID AB_561284), rabbit anti-phospho-STAT1 (Tyr-701) (D4A7; Cell Signaling Technology, Inc.; used for immunofluorescence; RRID AB_10950970), rabbit anti-phospho-STAT1 (Ser-727) (catalog no. 9177; Cell Signaling Technology, Inc.; RRID AB_2197983), and rabbit anti-phospho-IRF3 (Ser-396) (catalog no. 29047; Cell Signaling Technology, Inc.). Antiflavivirus group antigen D1-4G2-4-15 (ATCC HB-112 hybridoma; RRID CVCL_J890) and mouse anti-YFV ascites fluid were used in flavivirus focus-forming assays. Human and murine IFNs α4 and β were prepared in-house as described previously (36). Lipopolysaccharides (LPSs) from Escherichia coli 0111:B4 were purchased from Sigma. Poly(I:C) was purchased from R&D Systems.

### Treatment of cells with IFN-α/β, LPS, poly(I:C), and virus.

For experiments involving IFN or LPS treatment of cells, IFN-α/β (1:1 ratio of IFN-α4 to IFN-β; called IFN-α/β throughout) or LPS was applied directly to cells in medium prewarmed to room temperature or 30°C. IFN-α/β dosages selected for individual experiments represent the range of concentrations of IFN-α/β empirically determined to elicit robust ISG induction as well as observable dose-dependent antiarboviral activity. The required dosage, then, necessarily varied according to the cell type used, the IFN-α/β sensitivity of individual viruses, murine versus human, and the experimental aim. Dosages used for specific experiments are noted in the text and figure legends. LPS was used at a concentration of 250 ng/ml. After addition of the stimulus, cells were moved immediately to the appropriate temperature treatments, and the stimulus was left until sample collection. For experiments involving poly(I:C) treatment of cells, 50 µg poly(I:C) was transfected into MEF cells using the Neon transfection system (Invitrogen) at room temperature according to the manufacturer’s instructions and recommended settings. Pooled transfections were then divided among temperature conditions. For experiments involving virus infection at different temperatures, all infections were performed for 1 h at 30°C to minimize temperature-dependent variation in attachment and entry efficiency, unless otherwise noted. Cells were then washed in phosphate-buffered saline (PBS) and placed in fresh growth medium before being placed under different temperature conditions.

### Viral growth curves.

Vero, MEF, or primary murine osteoblast cells were treated for 12 to 16 h at 30, 37, or 39°C with 0 or 100 to 1,000 IU/ml human (Vero) or murine (MEF/osteoblast) IFN-α/β, rinsed in room-temperature PBS, and infected at a multiplicity of infection (MOI) of 0.1 as described above. Supernatants were harvested at the indicated time points, and viral growth was determined by plaque assay on BHK-21 cells for alphaviruses, by focus-forming assay on Vero cells for flaviviruses, and by plaque assay on Vero cells for RVFV. All quantification assays were performed at 37°C.

### Cytopathic effect (CPE) inhibition assays.

Vero cells were treated for 12 to 16 h at 30, 34, 37, or 39°C with 2-fold decreasing concentrations of human or mouse IFN-α/β in 96-well plates, beginning with 4,000 IU. Cells were then infected at an MOI of 10 and returned to the original temperature condition for 96 h. The concentration of IFN-α/β necessary to inhibit 50% of cell death was determined visually by crystal violet staining.

### Western blotting.

Protein lysates were taken in radioimmunoprecipitation assay (RIPA) buffer (50 mM Tris, pH 7.4, 150 mM NaCl, 1% NP-40, 0.5% sodium deoxycholate, 0.1% sodium dodecyl sulfate [SDS], 1 mM EDTA, 1 mM EGTA) supplemented with protease inhibitors (1 mM phenylmethylsulfonyl fluoride, 1 µg/ml leupeptin, 1 µg/ml pepstatin) and a phosphatase inhibitor cocktail (Sigma). Protein concentrations were determined by bicinchoninic acid (BCA) assay (Pierce) according to the manufacturer’s instructions. Equal amounts of protein (15 µg) per sample were resolved by SDS-PAGE on a 10% polyacrylamide gel, and proteins were transferred to a polyvinylidene difluoride (PVDF) membrane (Bio-Rad). Membranes were blocked in Tris-buffered saline with 0.1% Tween 20 (TBS-T) containing 5% milk for at least 1 h. Primary antibody was diluted in TBS-T containing 3% bovine serum albumin (BSA) and applied to membranes overnight at 4°C and then washed 4 times in TBS-T (15 min per wash) and replaced with horseradish peroxidase (HRP)-conjugated secondary antibody, diluted in a 2% milk–TBS-T solution. Secondary antibody was incubated for at least 1 h at room temperature or overnight at 4°C and then washed 4 times in TBS-T, as described above. Finally, membranes were exposed to Pierce ECL Western blotting substrate, and signal was captured on X-ray film (GE Healthcare). Densitometry analysis was performed using ImageJ software (NIH).

### Quantitative PCR and NanoString.

Total cellular RNA was isolated from samples taken in TRIzol reagent (Ambion) per the manufacturer’s protocol using 1-bromo-3-chloropropane (BCP) and isopropanol. For qRT-PCR, 10 µg of RNA per sample was reverse transcribed into cDNA using random hexamer primers and semiquantitative PCR was performed using the Sybr green method on an ABI 7900 real-time PCR machine (Applied Biosystems) using gene-specific primers. See Table S1 for a complete list of primer sequences. Threshold cycle (*C*_*T*_) values were normalized to 18S rRNA and compared using the ΔΔ*C*_*T*_ method (86). For NanoString, 100 ng RNA from each sample was subjected to fluorescent probe hybridization (see Table S2 for complete list of gene targets and probe sequences) and fluorescent bar codes corresponding to individual target mRNA molecules were counted automatically with an nCounter analysis system (NanoString Technologies). All mRNA quantification steps were performed by the Genomics Research Core at the University of Pittsburgh. Raw mRNA counts for each target gene underwent background subtraction and housekeeping gene normalization using nSolver 2.6 software (NanoString Technologies).

10.1128/mBio.00535-18.7 TABLE S1  Primers used for qRT-PCR. Download TABLE S1, PDF file, 0.4 MB. Copyright © 2018 Lane et al. 2018 Lane et al. This content is distributed under the terms of the Creative Commons Attribution 4.0 International license.

10.1128/mBio.00535-18.8 TABLE S2  List of NanoString target genes and probe locations. Download TABLE S2, PDF file, 0.4 MB. Copyright © 2018 Lane et al. 2018 Lane et al. This content is distributed under the terms of the Creative Commons Attribution 4.0 International license.

### Biological interferon assays.

Concentrations of biologically active murine IFN-α/β were measured by bioassay as previously described (87). Briefly, supernatants or serum samples (200 µl) were acidified to pH 2.0 with 2 N HCl overnight at 4°C and then neutralized to pH 7.4 using 2 N NaOH. Samples were serially diluted 2-fold across 96-well plates seeded with L929 cells and incubated at 37°C for 24 h. Cells were then infected with 3 × 10^4^ PFU per well of encephalomyocarditis virus (EMCV) and incubated for an additional 24 h at 37°C prior to fixation and staining with crystal violet. The concentration of IFN-α/β in each sample was calculated from the dilution required to protect 50% of cells from CPE, compared with a standard IFN-α/β dilution series of known concentrations.

### Immunofluorescence.

IFN-α/β- or poly(I:C)-treated cells seeded on glass microscope slides were fixed in 4% paraformaldehyde at 4°C for at least 1 h at the indicated times poststimulation. Cells were permeabilized in 100% methanol at −20°C for 10 min and blocked in blocking buffer (phosphate-buffered saline with 3% BSA and 0.1% Triton X-100) containing 10% normal serum corresponding to the species of the secondary antibody for at least 1 h at room temperature. Cells were then incubated overnight at 4°C with primary antibody diluted in blocking buffer, washed, and incubated with fluorescently labeled secondary antibody and 4′,6-diamidino-2-phenylindole (DAPI) nuclear stain for 1 h at room temperature. Images were acquired on a Nikon A1 confocal microscope or an Olympus CKX41 inverted epifluorescence microscope. Image analysis was performed in NIS Elements v4.51.00. The DAPI channel was used to create a binary mask that identified both the nuclear location and individual cells. The intensity of the STAT-p channel for all pixels within the nuclear mask was summed and then divided by the total number of pixels to produce the mean STAT-p signal per nucleus expressed as arbitrary fluorescence units (AFU). Mean STAT-p signal was plotted for individual cells.

### Protein radiolabel.

MEF cells were incubated at 30, 37, or 39°C for 1 or 12 h. At each time point, growth medium was replaced with labeling medium (DMEM lacking cysteine and methionine [Cellgro] supplemented with 1% fetal bovine serum [FBS] and 1% penicillin-streptomycin [Pen-Strep]), and cells were returned to their temperature conditions for 15 min. Supernatants were then replaced with labeling medium containing 100 µCi/ml [^35^S]Cys-Met (MP Biomedicals), and plates were returned to their temperature conditions for another 15 min. Cells were rinsed in PBS, and lysates were prepared with equal volumes of RIPA buffer. Twenty microliters per sample was resolved on an 8% SDS-PAGE gel, which was then fixed and dried to allow for autoradiographic signal capture of X-ray film (GE Healthcare). Gels were exposed for 7 days at −80°C, and resulting protein signal was quantified by densitometry analysis of all visible proteins in each lane using ImageJ software (NIH).

### Translation reporters.

Construction and production of host-mimic firefly luciferase-expressing mRNA translation reporter constructs have been described previously (88–90). MEFs or *Ifnar1*^*−/−*^ MEFs were transfected with 5 µg RNA reporter per reaction using the Neon transfection system (Invitrogen) according to the manufacturer’s instructions and recommended settings. Single transfections were divided among 30, 37, and 39°C temperature treatments for the appropriate time course, and cell lysates were collected in passive lysis buffer (Promega). Firefly luciferase activity was quantified using the dual-luciferase reporter assay kit (Promega), and light production was measured on an Orion microplate luminometer (Berthold). Relative light unit (RLU) values were normalized to protein concentration in each sample, as determined by bicinchoninic acid assay (Pierce). Data are expressed at RLU per microgram of protein.

### Mouse core temperature reduction and infection.

Six-week-old male and female C57BL/6 mice (Jackson Laboratories) and *Ifnar1*^−/−^ mice (bred in-house) were used for all experiments. For induction of metabolic torpor, mice were housed for 4.5 days in 24-h low red light before torpor was induced by food withdrawal. Nontorpid mice were also housed in red light but had access to food and water *ad libitum*. Food and normal light conditions (12-h light/12-h dark) were restored 48 h after food withdrawal. For pharmacological temperature reduction, mice were given 300 µl intraperitoneal injections of 1.5 to 2 mg/kg of body weight reserpine (U.S. Pharmacopeia standard; Sigma) dissolved in 0.5% acetic acid. Reserpine solutions were prepared fresh by first dissolving the powder in 100% glacial acetic acid and then diluting to the desired concentration in sterile double-distilled water (ddH_2_O). Final reserpine solutions underwent 0.22-µm filter sterilization before being injected into mice. Mice were given 0.5-mg/kg booster doses as needed (when subcutaneous scruff temperature exceeded 30°C) to maintain core temperature reduction throughout an experiment. Control mice were given equal volumes of sterile 0.5% acetic acid only. Isoflurane-anesthetized mice were infected with 1,000 PFU CHIKV-LR, CHIKV-LR-E2-79K, or nanoluciferase (nLuc)-TaV viruses (described in reference 45) in a 10-µl volume injected subcutaneously into the left rear footpad. For torpor experiments, infections took place 12 h after food withdrawal. For reserpine experiments, infections were done 3 h after reserpine treatment. All mice were weighed and monitored for disease signs daily. Musculoskeletal disease in infected feet was quantified every 12 to 24 hours as described previously (91).

On days 2 and 6 postinfection, groups of mice were sacrificed by isoflurane overdose and blood samples were collected by cardiac puncture before perfusion of tissues with PBS. Perfused tissues were extracted and homogenized, and viral load in each tissue was determined by plaque assay on BHK-21 cells. Serum was separated from whole-blood samples using Microtainer tubes (BD Biosciences), and IFN-α/β concentrations were determined by biological IFN-α/β assay, as described above.

### *In vivo* bioluminescence imaging.

Mice were anesthetized with a low-dose continuous stream of isoflurane using the Xenogen XGI-8 gas anesthesia system (Caliper Life Sciences). The nLuc substrate furimazine (Nano-Glo luciferase assay system; Promega) was diluted in sterile Dulbecco’s PBS (DPBS) with Ca^2+^ and Mg^2+^ (Corning) and injected subcutaneously in the scruff (5 µl substrate in 500-µl total volume per mouse) and allowed to circulate for 3 min. After 3 min, bioluminescence was imaged using an IVIS Spectrum CT instrument (PerkinElmer). Exposure time was determined automatically according to signal strength using Living Image acquisition software (PerkinElmer). Resulting pseudocolored images, normalized for exposure time, were marked with regions of interest (ROIs) around the hind feet of each mouse, and luciferase signal was quantitated as average radiance (photons per second per square centimeter per steradian) in each ROI using Living Image software (PerkinElmer). Mice were imaged daily for up to 7 days postinfection, beginning with a preinfection image.

### Quantification and statistical analysis.

Statistical significance for all experiments was determined using GraphPad Prism software. Results were declared statistically significant when *P* was less than or equal to 0.05, for all statistical tests employed across experiments. Specific tests used as well as demarcations of additional levels of significance are detailed in the figure legends. All experiments were performed with at least 2 replicate samples per group, and all experiments were performed at least twice to confirm results, except for the NanoString analysis, which was performed once, with select results being confirmed by qPCR. Data from single representative experiments are shown in the figures, and pooled data are presented as either means ± standard deviations (SD) or geometric mean ± geometric SD as appropriate.

## References

[1] Schneider WM, Chevillotte MD, Rice CM 2014 Interferon-stimulated genes: a complex web of host defenses. Annu Rev Immunol 32:513–545. doi:10.1146/annurev-immunol-032713-120231. 24555472 PMC4313732

[2] Ryman KD, Klimstra WB, Nguyen KB, Biron CA, Johnston RE 2000 Alpha/beta interferon protects adult mice from fatal Sindbis virus infection and is an important determinant of cell and tissue tropism. J Virol 74:3366–3378. doi:10.1128/JVI.74.7.3366-3378.2000. 10708454 PMC111838

[3] van den Broek MF, Müller U, Huang S, Zinkernagel RM, Aguet M 1995 Immune defence in mice lacking type I and/or type II interferon receptors. Immunol Rev 148:5–18. doi:10.1111/j.1600-065X.1995.tb00090.x. 8825279

[4] Schilte C, Couderc T, Chretien F, Sourisseau M, Gangneux N, Guivel-Benhassine F, Kraxner A, Tschopp J, Higgs S, Michault A, Arenzana-Seisdedos F, Colonna M, Peduto L, Schwartz O, Lecuit M, Albert ML 2010 Type I IFN controls Chikungunya virus via its action on nonhematopoietic cells. J Exp Med 207:429–442. doi:10.1084/jem.20090851. 20123960 PMC2822618

[5] Ryman KD, Meier KC, Gardner CL, Adegboyega PA, Klimstra WB 2007 Non-pathogenic Sindbis virus causes hemorrhagic fever in the absence of alpha/beta and gamma interferons. Virology 368:273–285. doi:10.1016/j.virol.2007.06.039. 17681583

[6] McFadden G, Mohamed MR, Rahman MM, Bartee E 2009 Cytokine determinants of viral tropism. Nat Rev Immunol 9:645–655. doi:10.1038/nri2623. 19696766 PMC4373421

[7] Honda K, Taniguchi T 2006 IRFs: master regulators of signalling by Toll-like receptors and cytosolic pattern-recognition receptors. Nat Rev Immunol 6:644–658. doi:10.1038/nri1900. 16932750

[8] Goubau D, Deddouche S, Reis e Sousa C 2013 Cytosolic sensing of viruses. Immunity 38:855–869. doi:10.1016/j.immuni.2013.05.007. 23706667 PMC7111113

[9] MacMicking JD 2012 Interferon-inducible effector mechanisms in cell-autonomous immunity. Nat Rev Immunol 12:367–382. doi:10.1038/nri3210. 22531325 PMC4150610

[10] Sadler AJ, Williams BR 2008 Interferon-inducible antiviral effectors. Nat Rev Immunol 8:559–568. doi:10.1038/nri2314. 18575461 PMC2522268

[11] García-Sastre A, Durbin RK, Zheng H, Palese P, Gertner R, Levy DE, Durbin JE 1998 The role of interferon in influenza virus tissue tropism. J Virol 72:8550–8558. 9765393 10.1128/jvi.72.11.8550-8558.1998 PMC110265

[12] Matsukawa T, Sessler DI, Sessler AM, Schroeder M, Ozaki M, Kurz A, Cheng C 1995 Heat flow and distribution during induction of general anesthesia. Anesthesiology 82:662–673. doi:10.1097/00000542-199503000-00008. 7879935

[13] Brajkovic D, Ducharme MB, Frim J 2001 Relationship between body heat content and finger temperature during cold exposure. J Appl Physiol 90:2445–2452. doi:10.1152/jappl.2001.90.6.2445. 11356812

[14] Garmel GM 2012 Fever in adults, p 375–392. *In* Mahadevan SV, Garmel GM (ed), An introduction to clinical emergency medicine, 2nd ed. Cambridge University Press, Cambridge, United Kingdom.

[15] Foxman EF, Storer JA, Fitzgerald ME, Wasik BR, Hou L, Zhao H, Turner PE, Pyle AM, Iwasaki A 2015 Temperature-dependent innate defense against the common cold virus limits viral replication at warm temperature in mouse airway cells. Proc Natl Acad Sci U S A 112:827–832. doi:10.1073/pnas.1411030112. 25561542 PMC4311828

[16] Boonarkart C, Suptawiwat O, Sakorn K, Puthavathana P, Auewarakul P 2017 Exposure to cold impairs interferon-induced antiviral defense. Arch Virol 162:2231–2237. doi:10.1007/s00705-017-3334-0. 28361289

[17] Ruiz-Gomez J, Sosa-Martinez J 1965 Virus multiplication and interferon production at different temperatures in adult mice infected with Coxsackie B1 virus. Arch Gesamte Virusforsch 17:295–299. doi:10.1007/BF01267913. 5882877

[18] Postic B, DeAngelis C, Breinig MK, Monto HO 1966 Effect of temperature of the induction of interferons by endotoxin and virus. J Bacteriol 91:1277–1281. 5929756 10.1128/jb.91.3.1277-1281.1966 PMC316024

[19] Hirai N, Hill NO, Osther K 1984 Temperature influences on different human alpha interferon activities. J Interferon Res 4:507–516. doi:10.1089/jir.1984.4.507. 6094682

[20] Letchworth GJ III, Carmichael LE 1984 The effect of temperature on production and function of bovine interferons. Arch Virol 82:211–221. doi:10.1007/BF01311164. 6210070

[21] Letchworth GJ, Carmichael LE 1984 Local tissue temperature: a critical factor in the pathogenesis of bovid herpesvirus 2. Infect Immun 43:1072–1079. 6199299 10.1128/iai.43.3.1072-1079.1984 PMC264296

[22] Groveman DS, Borden EC, Merritt JA, Robins HI, Steeves R, Bryan GT 1984 Augmented antiproliferative effects of interferons at elevated temperatures against human bladder carcinoma cell lines. Cancer Res 44:5517–5521. 6498814

[23] Fleischmann WR Jr, Fleischmann CM, Gindhart TD 1986 Effect of hyperthermia on the antiproliferative activities of murine α-, β-, and γ-interferon: differential enhancement of effect of hyperthermia on the antiproliferative activities of murine γ-interferon. Cancer Res 46:8–13. 2998611

[24] Gifford GE 1963 Effect of environmental changes upon antiviral action of interferon. Proc Soc Exp Biol Med 114:644–649. doi:10.3181/00379727-114-28757. 14120313

[25] Kirn A, Schieffner A, Tinland R 1967 Lack of correlation between production of interferon and protection of temperature in mice infected with Sindbis virus. Nature 215:86–87. doi:10.1038/215086a0. 6053416

[26] Lockart RZ Jr, Bayliss NL, Toy ST, Yin FH 1968 Viral events necessary for the induction of interferon in chick embryo cells. J Virol 2:962–965. 5723701 10.1128/jvi.2.10.962-965.1968 PMC375424

[27] Skehel JJ, Burke DC 1968 A temperature-sensitive event in interferon production. J Gen Virol 3:191–199. doi:10.1099/0022-1317-3-2-191. 5698681

[28] Cole GA, Wisseman CL 1969 The effect of hyperthermia on dengue virus infection of mice. Proc Soc Exp Biol Med 130:359–363. doi:10.3181/00379727-130-33555. 5765265

[29] Pusztai R, Béládi I, Bakay M, Mucsi I 1969 Effect of ultraviolet irradiation and heating on the interferon-inducing capacity of human adenoviruses. J Gen Virol 4:169–176. doi:10.1099/0022-1317-4-2-169. 5772486

[30] Ahl R 1970 Temperature-dependent interferon-sensitivity of foot-and-mouth disease virus. Arch Gesamte Virusforsch 32:163–170. doi:10.1007/BF01249952. 4322840

[31] Haahr S, Teisner B 1973 The influence of different temperatures on mortality, virus multiplication and interferon production in adult mice infected with Coxsackie B1 virus. Arch Gesamte Virusforsch 42:273–277. doi:10.1007/BF01265652. 4754181

[32] Murphy BR, Baron S, Chalhub EG, Uhlendorf CP, Chanock RM 1973 Temperature-sensitive mutants of influenza virus. IV. Induction of interferon in the nasopharynx by wild-type and a temperature-sensitive recombinant virus. J Infect Dis 128:488–493. doi:10.1093/infdis/128.4.488. 4743545

[33] Atkins GJ, Johnston MD, Westmacott LM, Burke DC 1974 Induction of interferon in chick cells by temperature-sensitive mutants of Sindbis virus. J Gen Virol 25:381–390. doi:10.1099/0022-1317-25-3-381. 4475093

[34] Atkins GJ, Lancashire CL 1976 The induction of interferon by temperature-sensitive mutants of Sindbis virus: its relationship to double-stranded RNA synthesis and cytopathic effect. J Gen Virol 30:157–165. doi:10.1099/0022-1317-30-2-157. 950559

[35] Stanton GJ, Langford MP, Baron S 1977 Effect of interferon, elevated temperature, and cell type on replication of acute hemorrhagic conjunctivitis viruses. Infect Immun 18:370–376. 200562 10.1128/iai.18.2.370-376.1977 PMC421242

[36] Yin J, Gardner CL, Burke CW, Ryman KD, Klimstra WB 2009 Similarities and differences in antagonism of neuron alpha/beta interferon responses by Venezuelan equine encephalitis and Sindbis alphaviruses. J Virol 83:10036–10047. doi:10.1128/JVI.01209-09. 19641001 PMC2748036

[37] Bhalla N, Sun C, Metthew Lam LK, Gardner CL, Ryman KD, Klimstra WB 2016 Host translation shutoff mediated by non-structural protein 2 is a critical factor in the antiviral state resistance of Venezuelan equine encephalitis virus. Virology 496:147–165. doi:10.1016/j.virol.2016.06.005. 27318152 PMC5821108

[38] Verbruggen P, Ruf M, Blakqori G, Överby AK, Heidemann M, Eick D, Weber F 2011 Interferon antagonist NSs of la Crosse virus triggers a DNA damage response-like degradation of transcribing RNA polymerase II. J Biol Chem 286:3681–3692. doi:10.1074/jbc.M110.154799. 21118815 PMC3030371

[39] Branca AA, Faltynek CR, D’Alessandro SB, Baglioni C 1982 Interaction of interferon with cellular receptors. Internalization and degradation of cell-bound interferon. J Biol Chem 257:13291–13296. 6292184

[40] Platanias LC 2005 Mechanisms of type-I- and type-II-interferon-mediated signalling. Nat Rev Immunol 5:375–386. doi:10.1038/nri1604. 15864272

[41] Gardner J, Anraku I, Le TT, Larcher T, Major L, Roques P, Schroder WA, Higgs S, Suhrbier A 2010 Chikungunya virus arthritis in adult wild-type mice. J Virol 84:8021–8032. doi:10.1128/JVI.02603-09. 20519386 PMC2916516

[42] Himms-Hagen J 1985 Food restriction increases torpor and improves brown adipose tissue thermogenesis in ob/ob mice. Am J Physiol 248:E531–E539. doi:10.1152/ajpendo.1985.248.5.E531. 4039535

[43] Cox B, Tha SJ 1975 The role of dopamine and noradrenaline in temperature control of normal and reserpine-pretreated mice. J Pharm Pharmacol 27:242–247. doi:10.1111/j.2042-7158.1975.tb10693.x. 239116

[44] Zeisberger E 1987 The roles of monoaminergic neurotransmitters in thermoregulation. Can J Physiol Pharmacol 65:1395–1401. doi:10.1139/y87-219. 2887271

[45] Sun C, Gardner CL, Watson AD, Ryman KD, Klimstra WB 2014 Stable, high-level expression of reporter proteins from improved alphavirus expression vectors to track replication and dissemination during encephalitic and arthritogenic disease. J Virol 88:2035–2046. doi:10.1128/JVI.02990-13. 24307590 PMC3911548

[46] Russwurm S, Stonāns I, Schwerter K, Stonāne E, Meissner W, Reinhart K 2002 Direct influence of mild hypothermia on cytokine expression and release in cultures of human peripheral blood mononuclear cells. J Interferon Cytokine Res 22:215–221. doi:10.1089/107999002753536185. 11911804

[47] Fairchild KD, Singh IS, Patel S, Drysdale BE, Viscardi RM, Hester L, Lazusky HM, Hasday JD 2004 Hypothermia prolongs activation of NF-κΒ and augments generation of inflammatory cytokines. Am J Physiol Cell Physiol 287:C422–C431. doi:10.1152/ajpcell.00507.2003. 15070815

[48] Hagiwara S, Iwasaka H, Matsumoto S, Noguchi T 2007 Changes in cell culture temperature alter release of inflammatory mediators in murine macrophagic RAW264.7 cells. Inflamm Res 56:297–303. doi:10.1007/s00011-007-6161-z. 17659435

[49] Matsui T, Motoki Y, Yoshida Y 2013 Hypothermia reduces toll-like receptor 3-activated microglial interferon-beta and nitric oxide production. Mediators Inflamm 2013:436263. doi:10.1155/2013/436263. 23589665 PMC3621171

[50] Du G, Liu Y, Li J, Liu W, Wang Y, Li H 2013 Hypothermic microenvironment plays a key role in tumor immune subversion. Int Immunopharmacol 17:245–253. doi:10.1016/j.intimp.2013.06.018. 23831011

[51] Foxman EF, Storer JA, Vanaja K, Levchenko A, Iwasaki A 2016 Two interferon-independent double-stranded RNA-induced host defense strategies suppress the common cold virus at warm temperature. Proc Natl Acad Sci U S A 113:8496–8501. doi:10.1073/pnas.1601942113. 27402752 PMC4968739

[52] Smale ST, Plevy SE, Weinmann AS, Zhou L, Ramirez-Carrozzi VR, Pope SD, Bhatt DM, Tong AJ 2013 Toward an understanding of the gene-specific and global logic of inducible gene transcription. Cold Spring Harb Symp Quant Biol 78:61–68. doi:10.1101/sqb.2013.78.020313. 24747344

[53] Sumitomo Y, Higashitsuji H, Higashitsuji H, Liu Y, Fujita T, Sakurai T, Candeias MM, Itoh K, Chiba T, Fujita J 2012 Identification of a novel enhancer that binds Sp1 and contributes to induction of cold-inducible RNA-binding protein (cirp) expression in mammalian cells. BMC Biotechnol 12:72. doi:10.1186/1472-6750-12-72. 23046908 PMC3534229

[54] Au-Yeung N, Mandhana R, Horvath CM 2013 Transcriptional regulation by STAT1 and STAT2 in the interferon JAK-STAT pathway. JAKSTAT 2:e23931. doi:10.4161/jkst.23931. 24069549 PMC3772101

[55] Chang HM, Paulson M, Holko M, Rice CM, Williams BRG, Marié I, Levy DE 2004 Induction of interferon-stimulated gene expression and antiviral responses require protein deacetylase activity. Proc Natl Acad Sci U S A 101:9578–9583. doi:10.1073/pnas.0400567101. 15210966 PMC470717

[56] Kadota S, Nagata K 2014 Silencing of IFN-stimulated gene transcription is regulated by histone H1 and its chaperone TAF-I. Nucleic Acids Res 42:7642–7653. doi:10.1093/nar/gku485. 24878923 PMC4081089

[57] Medzhitov R, Horng T 2009 Transcriptional control of the inflammatory response. Nat Rev Immunol 9:692–703. doi:10.1038/nri2634. 19859064

[58] Vilcek J, Havell EA 1973 Stabilization of interferon messenger RNA activity by treatment of cells with metabolic inhibitors and lowering of the incubation temperature. Proc Natl Acad Sci U S A 70:3909–3913. doi:10.1073/pnas.70.12.3909. 4544057 PMC427355

[59] Al-Fageeh MB, Smales CM 2006 Control and regulation of the cellular responses to cold shock: the responses in yeast and mammalian systems. Biochem J 397:247–259. doi:10.1042/BJ20060166. 16792527 PMC1513281

[60] Roobol A, Carden MJ, Newsam RJ, Smales CM 2009 Biochemical insights into the mechanisms central to the response of mammalian cells to cold stress and subsequent rewarming. FEBS J 276:286–302. doi:10.1111/j.1742-4658.2008.06781.x. 19054067

[61] Knight JR, Bastide A, Roobol A, Roobol J, Jackson TJ, Utami W, Barrett DA, Smales CM, Willis AE 2015 Eukaryotic elongation factor 2 kinase regulates the cold stress response by slowing translation elongation. Biochem J 465:227–238. doi:10.1042/BJ20141014. 25353634

[62] Aguilar PV, Adams AP, Wang E, Kang W, Carrara AS, Anishchenko M, Frolov I, Weaver SC 2008 Structural and nonstructural protein genome regions of eastern equine encephalitis virus are determinants of interferon sensitivity and murine virulence. J Virol 82:4920–4930. doi:10.1128/JVI.02514-07. 18353963 PMC2346730

[63] Garmashova N, Atasheva S, Kang W, Weaver SC, Frolova E, Frolov I 2007 Analysis of Venezuelan equine encephalitis virus capsid protein function in the inhibition of cellular transcription. J Virol 81:13552–13565. doi:10.1128/JVI.01576-07. 17913819 PMC2168819

[64] Simmons JD, White LJ, Morrison TE, Montgomery SA, Whitmore AC, Johnston RE, Heise MT 2009 Venezuelan equine encephalitis virus disrupts STAT1 signaling by distinct mechanisms independent of host shutoff. J Virol 83:10571–10581. doi:10.1128/JVI.01041-09. 19656875 PMC2753124

[65] Fros JJ, Liu WJ, Prow NA, Geertsema C, Ligtenberg M, Vanlandingham DL, Schnettler E, Vlak JM, Suhrbier A, Khromykh AA, Pijlman GP 2010 Chikungunya virus nonstructural protein 2 inhibits type I/II interferon-stimulated JAK-STAT signaling. J Virol 84:10877–10887. doi:10.1128/JVI.00949-10. 20686047 PMC2950581

[66] Ramseur JM, Friedman RM 1977 Prolonged infection of interferon-treated cells by vesicular stomatitis virus: possible role of temperature-sensitive mutants and interferon. J Gen Virol 37:523–533. doi:10.1099/0022-1317-37-3-523.

[67] Barrett PN, Atkins GJ 1981 Establishment of persistent infection in mouse cells by Sindbis virus and its temperature-sensitive mutants. J Gen Virol 54:57–65. doi:10.1099/0022-1317-54-1-57. 7288408

[68] Chaloner Larsson G, Johnson-Lussenburg CM 1981 Establishment and maintenance of a persistent infection of L132 cells by human coronavirus strain 229E. Arch Virol 69:117–129. doi:10.1007/BF01315155. 6171237 PMC7086901

[69] Cunningham AL, Fraser JR 1985 Persistent rubella virus infection of human synovial cells cultured in vitro. J Infect Dis 151:638–645. doi:10.1093/infdis/151.4.638. 3973414

[70] Gercel C, Mahan KB, Hamparian VV 1985 Preliminary characterization of a persistent infection of HeLa cells with human rhinovirus type 2. J Gen Virol 66:131–139. doi:10.1099/0022-1317-66-1-131. 2578551

[71] Joguet G, Mansuy JM, Matusali G, Hamdi S, Walschaerts M, Pavili L, Guyomard S, Prisant N, Lamarre P, Dejucq-Rainsford N, Pasquier C, Bujan L 2017 Effect of acute Zika virus infection on sperm and virus clearance in body fluids: a prospective observational study. Lancet Infect Dis 17:1200–1208. doi:10.1016/S1473-3099(17)30444-9. 28838639

[72] Biava M, Caglioti C, Castilletti C, Bordi L, Carletti F, Colavita F, Quartu S, Nicastri E, Iannetta M, Vairo F, Liuzzi G, Taglietti F, Ippolito G, Capobianchi MR, Lalle E 2018 Persistence of ZIKV-RNA in the cellular fraction of semen is accompanied by a surrogate-marker of viral replication. Diagnostic implications for sexual transmission. New Microbiol 41:30–33. 29112766

[73] García-Bujalance S, Gutiérrez-Arroyo A, De la Calle F, Díaz-Menéndez M, Arribas JR, García-Rodríguez J, Arsuaga M 2017 Persistence and infectivity of Zika virus in semen after returning from endemic areas: report of 5 cases. J Clin Virol 96:110–115. doi:10.1016/j.jcv.2017.10.006. 29053990

[74] Hoarau JJ, Jaffar Bandjee MC, Krejbich Trotot P, Das T, Li-Pat-Yuen G, Dassa B, Denizot M, Guichard E, Ribera A, Henni T, Tallet F, Moiton MP, Gauzère BA, Bruniquet S, Jaffar Bandjee Z, Morbidelli P, Martigny G, Jolivet M, Gay F, Grandadam M, Tolou H, Vieillard V, Debré P, Autran B, Gasque P 2010 Persistent chronic inflammation and infection by Chikungunya arthritogenic alphavirus in spite of a robust host immune response. J Immunol 184:5914–5927. doi:10.4049/jimmunol.0900255. 20404278

[75] Burke CW, Gardner CL, Steffan JJ, Ryman KD, Klimstra WB 2009 Characteristics of alpha/beta interferon induction after infection of murine fibroblasts with wild-type and mutant alphaviruses. Virology 395:121–132. doi:10.1016/j.virol.2009.08.039. 19782381 PMC4381786

[76] Gardner CL, Burke CW, Tesfay MZ, Glass PJ, Klimstra WB, Ryman KD 2008 Eastern and Venezuelan equine encephalitis viruses differ in their ability to infect dendritic cells and macrophages: impact of altered cell tropism on pathogenesis. J Virol 82:10634–10646. doi:10.1128/JVI.01323-08. 18768986 PMC2573165

[77] Klimstra WB, Ryman KD, Johnston RE 1998 Adaptation of Sindbis virus to BHK cells selects for use of heparan sulfate as an attachment receptor. J Virol 72:7357–7366. 9696832 10.1128/jvi.72.9.7357-7366.1998 PMC109960

[78] Tsetsarkin K, Higgs S, McGee CE, De Lamballerie X, Charrel RN, Vanlandingham DL 2006 Infectious clones of Chikungunya virus (La Reunion isolate) for vector competence studies. Vector Borne Zoonotic Dis 6:325–337. doi:10.1089/vbz.2006.6.325. 17187566

[79] Anishchenko M, Paessler S, Greene IP, Aguilar PV, Carrara AS, Weaver SC 2004 Generation and characterization of closely related epizootic and enzootic infectious cDNA clones for studying interferon sensitivity and emergence mechanisms of Venezuelan equine encephalitis virus. J Virol 78:1–8. doi:10.1128/JVI.78.1.1-8.2004. 14671082 PMC303380

[80] Frolov I, Agapov E, Hoffman TA Jr, Prágai BM, Lippa M, Schlesinger S, Rice CM 1999 Selection of RNA replicons capable of persistent noncytopathic replication in mammalian cells. J Virol 73:3854–3865. 10196280 10.1128/jvi.73.5.3854-3865.1999 PMC104163

[81] Gardner CL, Hritz J, Sun C, Vanlandingham DL, Song TY, Ghedin E, Higgs S, Klimstra WB, Ryman KD 2014 Deliberate attenuation of Chikungunya virus by adaptation to heparan sulfate-dependent infectivity: a model for rational arboviral vaccine design. PLoS Negl Trop Dis 8:e2719. doi:10.1371/journal.pntd.0002719. 24587470 PMC3930508

[82] Davis NL, Willis LV, Smith JF, Johnston RE 1989 In vitro synthesis of infectious Venezuelan equine encephalitis virus RNA from a cDNA clone: analysis of a viable deletion mutant. Virology 171:189–204. doi:10.1016/0042-6822(89)90526-6. 2525837

[83] Watson AM, Lam LK, Klimstra WB, Ryman KD 2016 The 17D-204 vaccine strain-induced protection against virulent yellow fever virus is mediated by humoral immunity and CD4+ but not CD8+ T cells. PLoS Pathog 12:e1005786. doi:10.1371/journal.ppat.1005786. 27463517 PMC4962991

[84] Bird BH, Albariño CG, Nichol ST 2007 Rift Valley fever virus lacking NSm proteins retains high virulence in vivo and may provide a model of human delayed onset neurologic disease. Virology 362:10–15. doi:10.1016/j.virol.2007.01.046. 17412386

[85] Caroline AL, Powell DS, Bethel LM, Oury TD, Reed DS, Hartman AL 2014 Broad spectrum antiviral activity of favipiravir (T-705): protection from highly lethal inhalational Rift Valley fever. PLoS Neglect Trop Dis 8:e2790. doi:10.1371/journal.pntd.0002790. PMC3983105 24722586

[86] Livak KJ, Schmittgen TD 2001 Analysis of relative gene expression data using real-time quantitative PCR and the 2(-Delta Delta C(T)) method. Methods 25:402–408. doi:10.1006/meth.2001.1262. 11846609

[87] Trgovcich J, Aronson JF, Johnston RE 1996 Fatal Sindbis virus infection of neonatal mice in the absence of encephalitis. Virology 224:73–83. doi:10.1006/viro.1996.0508. 8862401

[88] Bick MJ, Carroll JW, Gao G, Goff SP, Rice CM, MacDonald MR 2003 Expression of the zinc finger antiviral protein inhibits alphavirus replication. J Virol 77:11555–11562. doi:10.1128/JVI.77.21.11555-11562.2003. 14557641 PMC229374

[89] Ryman KD, Meier KC, Nangle EM, Ragsdale SL, Korneeva NL, Rhoads RE, MacDonald MR, Klimstra WB 2005 Sindbis virus translation is inhibited by a PKR/RNase L-independent effector induced by alpha/beta interferon priming of dendritic cells. J Virol 79:1487–1499. doi:10.1128/JVI.79.3.1487-1499.2005. 15650175 PMC544143

[90] Tesfay MZ, Yin J, Gardner CL, Khoretonenko MV, Korneeva NL, Rhoads RE, Ryman KD, Klimstra WB 2008 Alpha/beta interferon inhibits cap-dependent translation of viral but not cellular mRNA by a PKR-independent mechanism. J Virol 82:2620–2630. doi:10.1128/JVI.01784-07. 18160435 PMC2259014

[91] Gardner CL, Burke CW, Higgs ST, Klimstra WB, Ryman KD 2012 Interferon-alpha/beta deficiency greatly exacerbates arthritogenic disease in mice infected with wild-type Chikungunya virus but not with the cell culture-adapted live-attenuated 181/25 vaccine candidate. Virology 425:103–112. doi:10.1016/j.virol.2011.12.020. 22305131 PMC3431213

[92] National Research Council 2011 Guide for the care and use of laboratory animals, 8th ed. The National Academies Press, Washington, DC. doi:10.17226/12910.

