# Supplementary material for: The Efficacy of the Interferon Alpha/Beta Response versus Arboviruses Is Temperature Dependent
Source: mBio. 2018 Apr 24;9(2):e00535-18. doi: 10.1128/mBio.00535-18 (PMC5915735; doi:10.1128/mBio.00535-18)
Supplement: FIG S1 [file mbo002183831sf1.pdf]

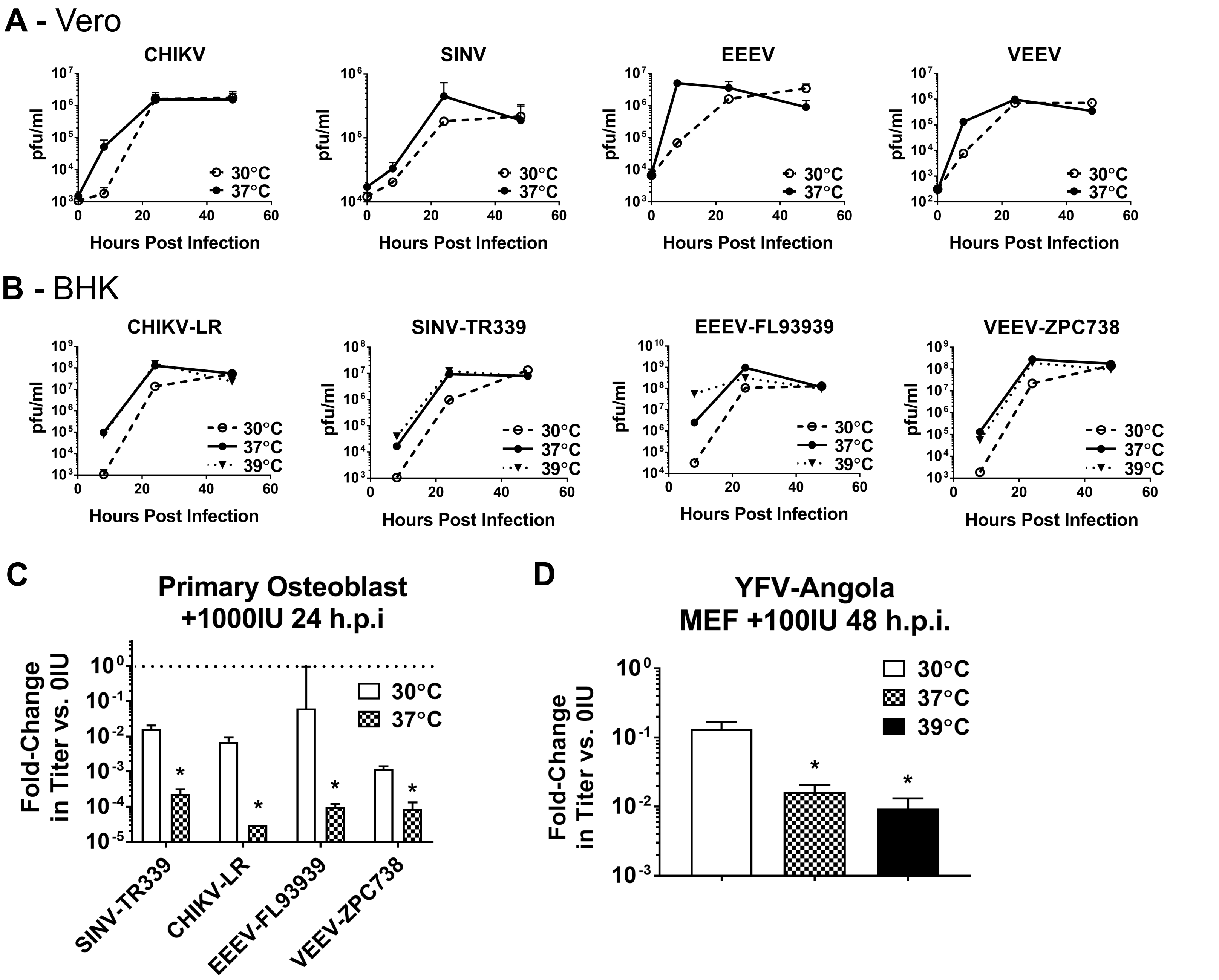

Figure S1: Efficacy of type I IFN against arboviruses is reduced at subnormal temperatures. Related to Figure 1.

A-B: Unprimed Vero (A) or baby hamster kidney (BHK; B) cells were infected in triplicate with the indicated alphaviruses at a M.O.I. of 0.1 and incubated at 30, 37, or 39°C. At the indicated times post infection, supernatants were harvested and viral titer was determined by plaque assay on BHK cells at 37°C. **C:** Osteoblasts cultured from adult CD-1 mice were treated with 1000 IU/mL IFN-α/β at 30 or 37°C overnight and then infected at a M.O.I. of 0.1 with the indicated alphaviruses. 24 h.p.i. supernatants were assessed for viral titer by plaque assay at 37°C. Data are expressed as fold-change in titer between IFN-α/β-treated and untreated cells at each temperature. *$p<0.001$ using multiple two-tailed *t* tests with Holm-Sidak correction of log-transformed fold-change values. **D:** MEFs were treated overnight with 100 IU/mL IFN-α/β at 30, 37, or 39°C and infected with YFV-Angola at M.O.I. 0.1. 48 h.p.i. supernatants were assayed for viral titer by focus-forming assay on Vero cells at 37°C. Data are presented as fold-change in titer between IFN-α/β-treated and untreated cells at each temperature. *$p<0.0001$ one-way ANOVA with Tukey's multiple comparison test of log-transformed fold-change values.
