# Supplementary material for: The Efficacy of the Interferon Alpha/Beta Response versus Arboviruses Is Temperature Dependent
Source: mBio. 2018 Apr 24;9(2):e00535-18. doi: 10.1128/mBio.00535-18 (PMC5915735; doi:10.1128/mBio.00535-18)
Supplement: FIG S2 [file mbo002183831sf2.pdf]

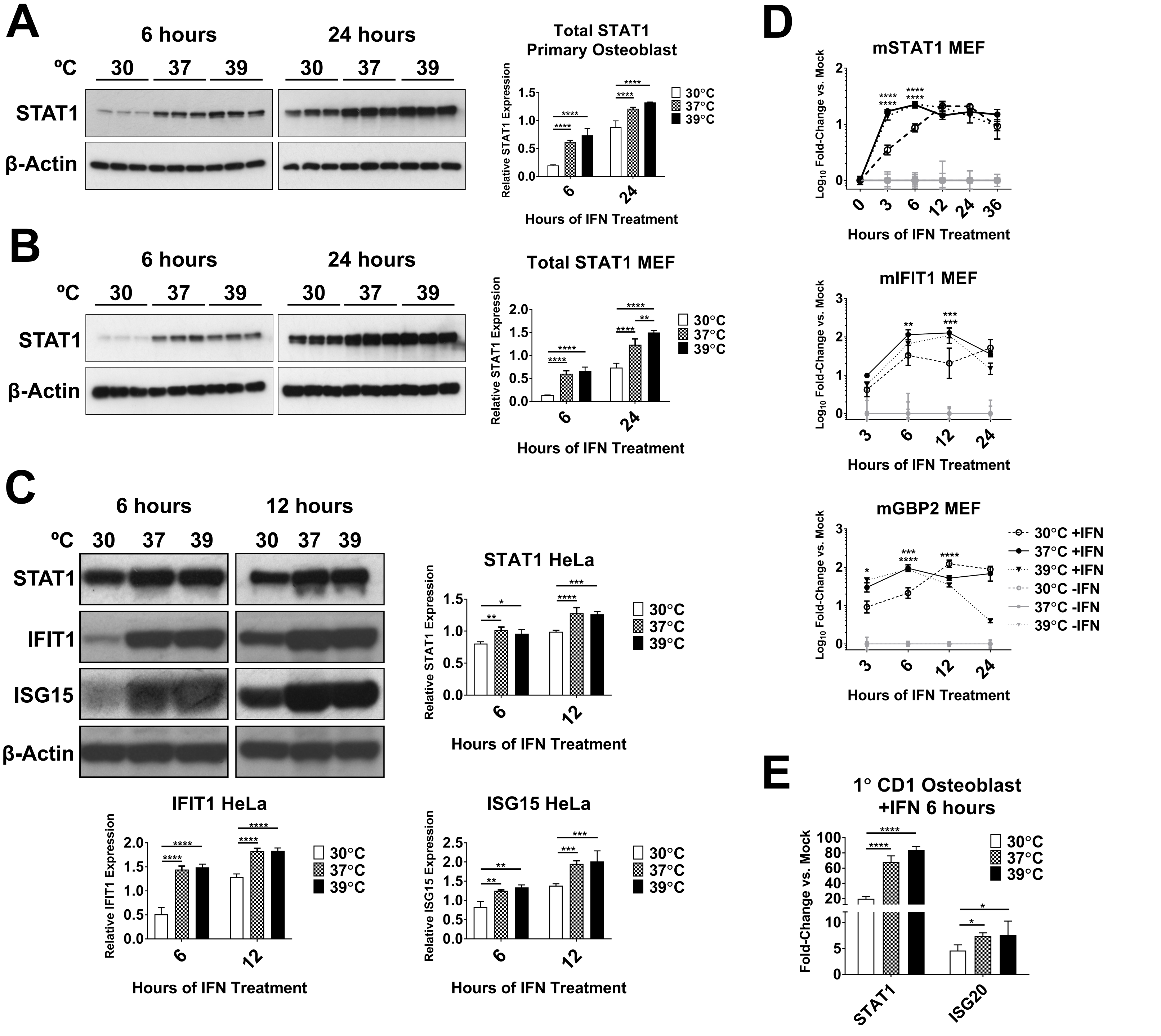

Figure S2: At subnormal temperatures, ISG protein and mRNA levels are reduced. Related to Figure 2.
A-C: Primary CD-1 osteoblasts (A), MEFs (B), and HeLa cells (C) were treated with 100 IU/mL IFN-α/β at 30, 37, or 39°C for 6-24 hours. Lysates were analyzed for ISG protein production by immunoblot. Graphs display densitometry analysis of ISG bands at each temperature normalized to β-actin. **D-E:** The procedure from (A-C) was repeated and total cellular RNA was probed for ISG mRNA content by qRT-PCR. Data are presented as $\log_{10}$ fold-change (D) or fold-change (E) between 18S-normalized Ct values for IFN-α/β-treated and untreated cells at each temperature. Statistics: *p<0.05, **p<0.01, ***p<0.001, ****p<0.0001, ns not significant by two-way ANOVA with Tukey's multiple comparison test.
