# Supplementary material for: The Efficacy of the Interferon Alpha/Beta Response versus Arboviruses Is Temperature Dependent
Source: mBio. 2018 Apr 24;9(2):e00535-18. doi: 10.1128/mBio.00535-18 (PMC5915735; doi:10.1128/mBio.00535-18)
Supplement: FIG S3 [file mbo002183831sf3.pdf]

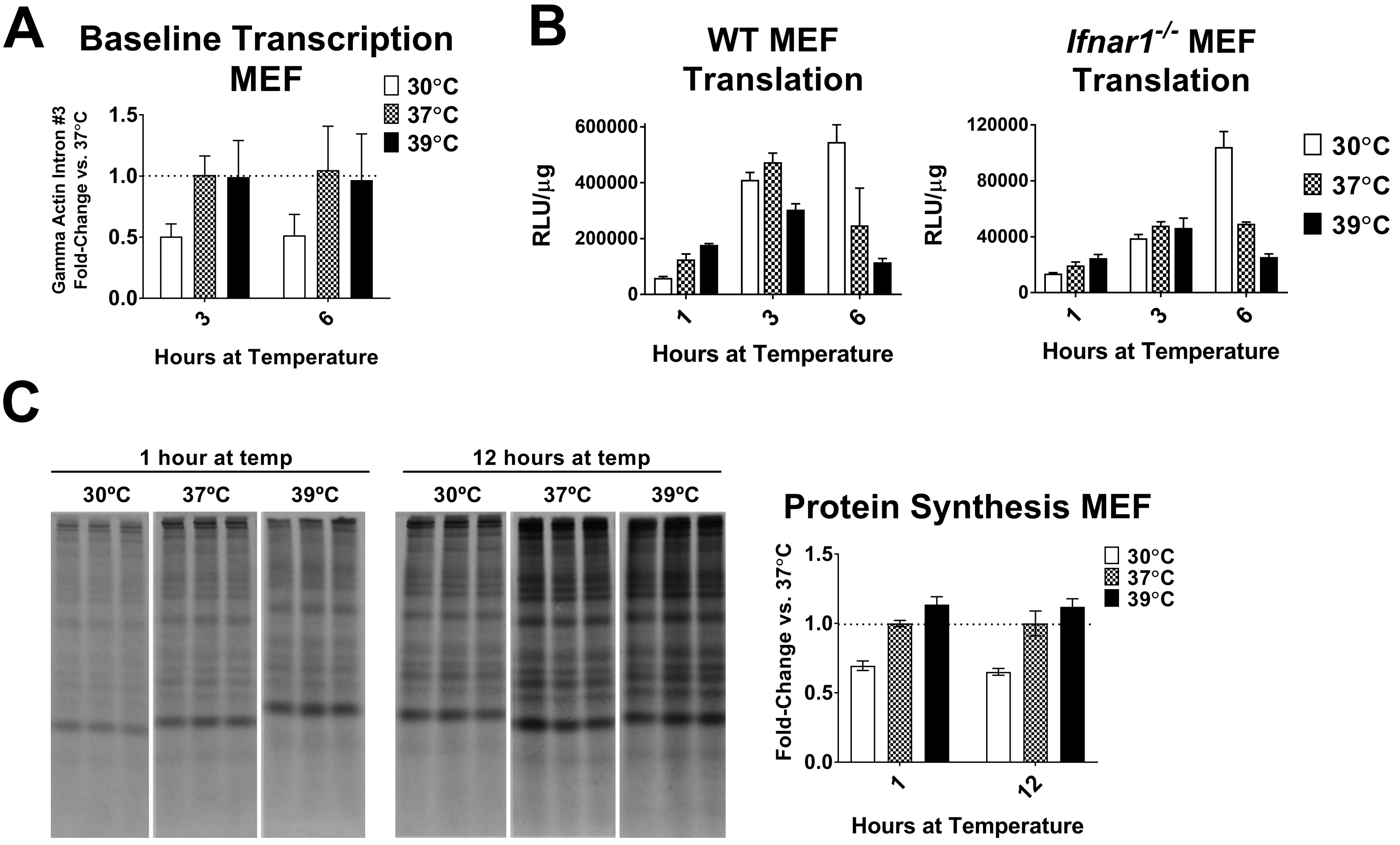

Figure S3: Temperature variation affects baseline transcription and translation rates.
A: Total cellular RNA from MEFs incubated at different temperatures were assayed for gamma actin intron #3 using qRT-PCR. Data are presented as fold-change in 18S-normalized Ct values versus 37°C. **B:** Wild-type or *Ifnar1*$^{-/-}$ MEF cells were transfected with 5 µg of *in vitro*-transcribed firefly luciferase-expressing reporter RNA and divided among 30, 37, and 39°C temperature conditions for 1, 3, or 6 hours. Luciferase translation efficiency was quantified by luciferase activity assay and RLU values were normalized to total protein content determined by BCA assay. **C:** MEF cells were incubated at 30, 37, or 39°C for 1 or 12 hours and new protein production was marked by [^{35}S]-labeled cysteine and methionine incorporation. Lysates were separated using SDS-PAGE and total protein was quantified using autoradiography followed by densitometry analysis of all visible bands in each lane. Graph: Quantification of (C). Data are presented as fold-change versus mean protein content at 37°C at each time point.
