# Supplementary material for: The Efficacy of the Interferon Alpha/Beta Response versus Arboviruses Is Temperature Dependent
Source: mBio. 2018 Apr 24;9(2):e00535-18. doi: 10.1128/mBio.00535-18 (PMC5915735; doi:10.1128/mBio.00535-18)
Supplement: FIG S4 [file mbo002183831sf4.pdf]

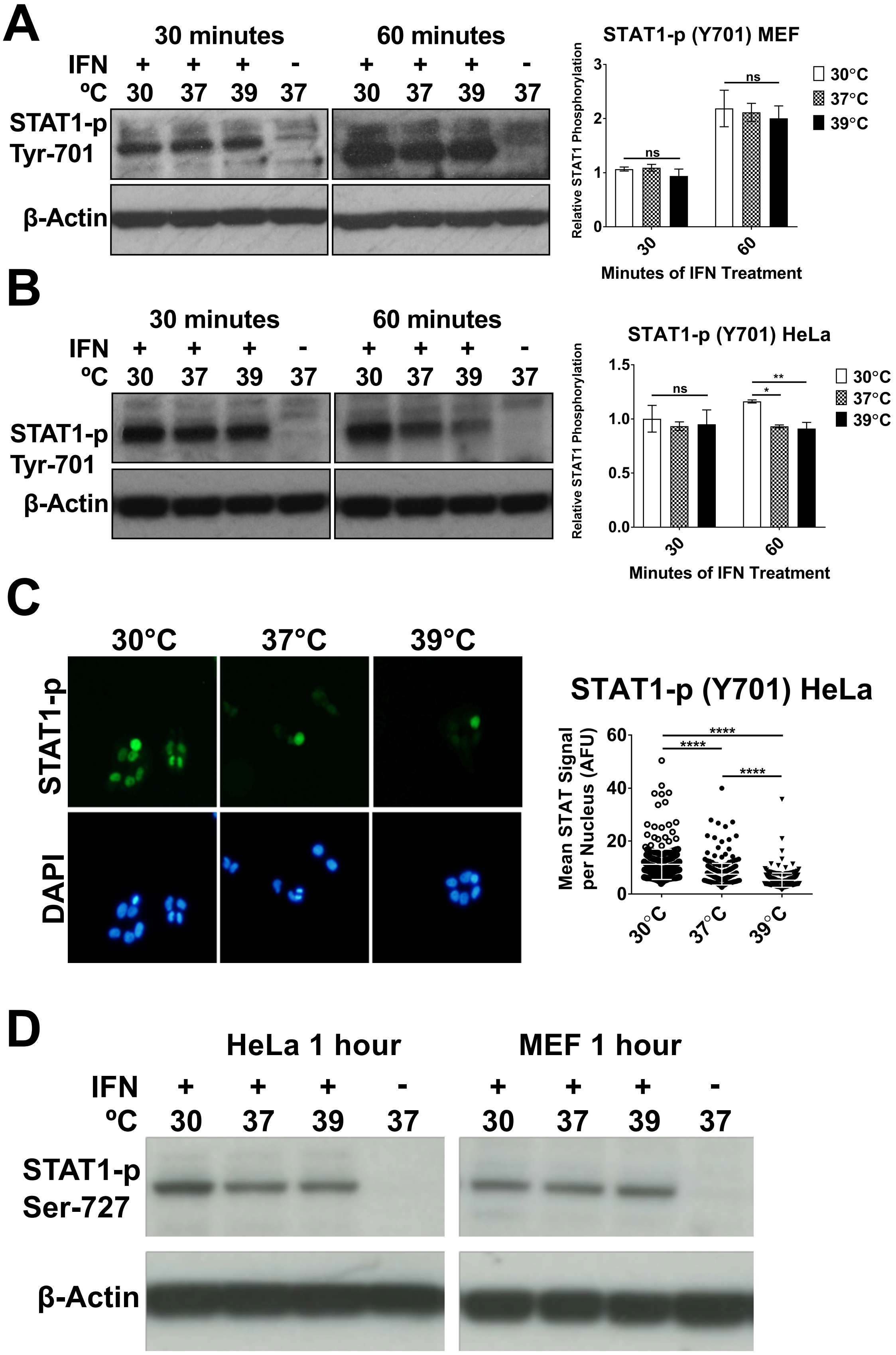

Figure S4: Suppression of type I IFN signaling pathways is not associated with early temperature effects. Related to Figure 3.

A, B, and D: MEF and HeLa cells were treated with 100 IU/mL IFN-α/β for 30 or 60 minutes at 30, 37, or 39°C and lysates were probed for STAT1 phosphorylated at Tyr-701 (A-B) or Ser-727 (D) by immunoblot. Graphs display densitometry analysis of STAT1-p bands normalized to β-actin at each temperature. Statistics for A and B: *p<0.05, **p<0.01, ***p<0.001, ****p<0.0001, ns not significant by two-way ANOVA with Tukey's multiple comparison test. **C:** HeLa cells treated with 1000 IU/mL IFN-α/β for 30 minutes at 30, 37, or 39°C were subjected to immunocytochemistry staining for STAT1-p (Y701). Confocal imaging shows phosphorylated STAT1 nuclear translocation. Graph displays average nuclear STAT1-p signal intensity per imaged nuclear area at each temperature. ****p<0.0001 one-way ANOVA with Tukey's multiple comparison test.
