# Supplementary material for: The Efficacy of the Interferon Alpha/Beta Response versus Arboviruses Is Temperature Dependent
Source: mBio. 2018 Apr 24;9(2):e00535-18. doi: 10.1128/mBio.00535-18 (PMC5915735; doi:10.1128/mBio.00535-18)
Supplement: FIG S5 [file mbo002183831sf5.pdf]

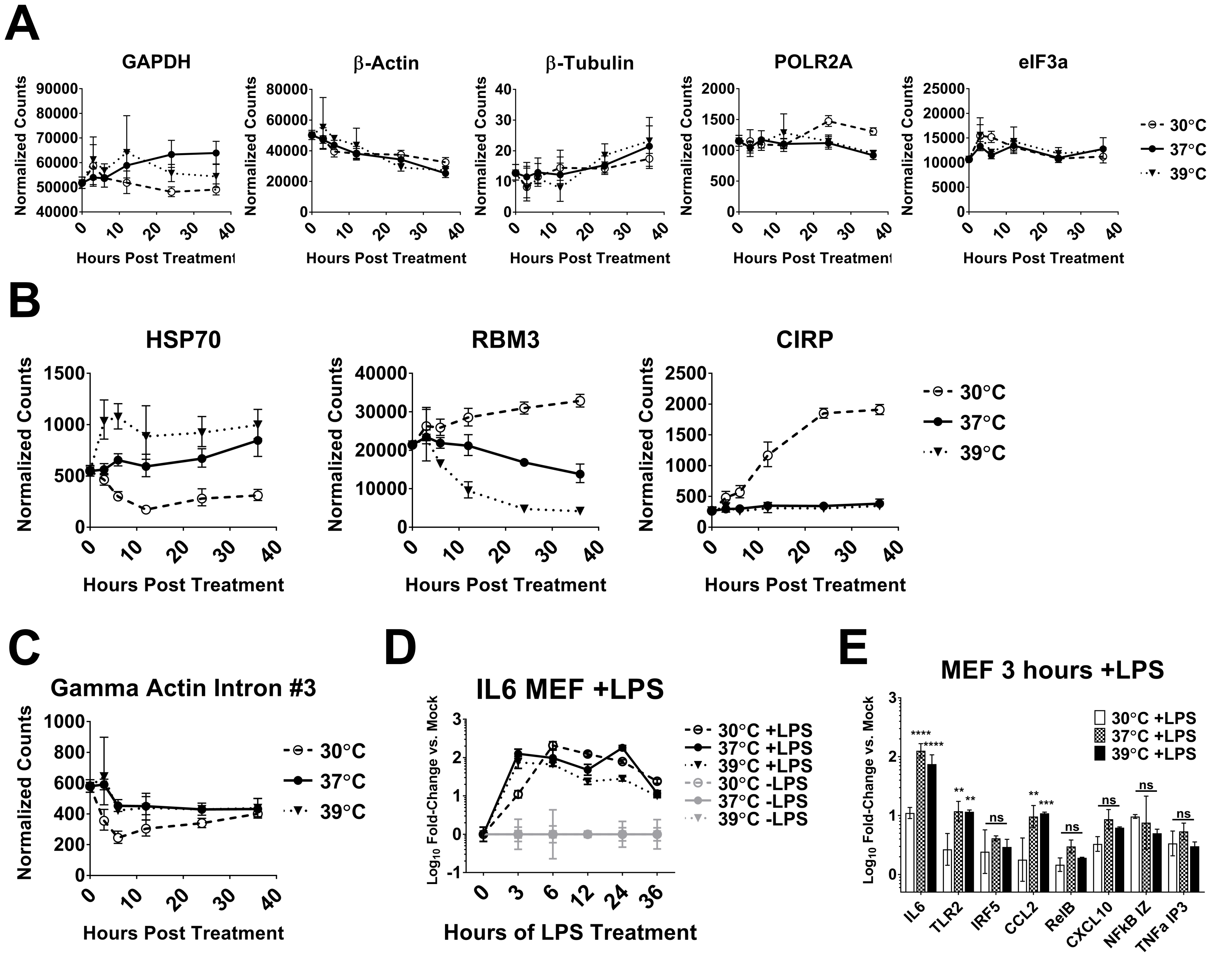

Figure S5: NanoString analysis detects temperature-sensitive gene transcription. Related to Figure 4.
MEF cells were treated in with 100IU IFN-α/β or 250 ng/mL LPS, or were left untreated, at 30, 37, or 39°C for the time course indicated in duplicate samples. At each time point, total cellular RNA was harvested and subjected to direct mRNA quantification of 125 gene targets (see Table S2) using the NanoString platform. Raw mRNA counts in each sample were subject to background subtraction followed by normalization to the geometric mean of five normalization genes (B-actin, GAPDH, B-Tubulin, POLR2A, and eIF3a) in the same sample. **A:** Background-subtracted mRNA count values of the five genes used for normalization, with IFN-α/β- or LPS-stimulated and unstimulated samples pooled at each temperature. **B:** Normalized expression profile of three known temperature-responsive genes, with IFN-α/β- or LPS-stimulated and unstimulated samples pooled at each temperature. **C:** Gamma actin intron #3 expression. **D:** IL6 expression in LPS-treated MEFs in an independent experiment, quantified by qRT-PCR. **E:** Verification of expression of other LPS-responsive genes in MEFs at 3 hours post-LPS treatment in an independent experiment, quantified by qRT-PCR.
