# Supplementary material for: The Efficacy of the Interferon Alpha/Beta Response versus Arboviruses Is Temperature Dependent
Source: mBio. 2018 Apr 24;9(2):e00535-18. doi: 10.1128/mBio.00535-18 (PMC5915735; doi:10.1128/mBio.00535-18)
Supplement: FIG S6 [file mbo002183831sf6.pdf]

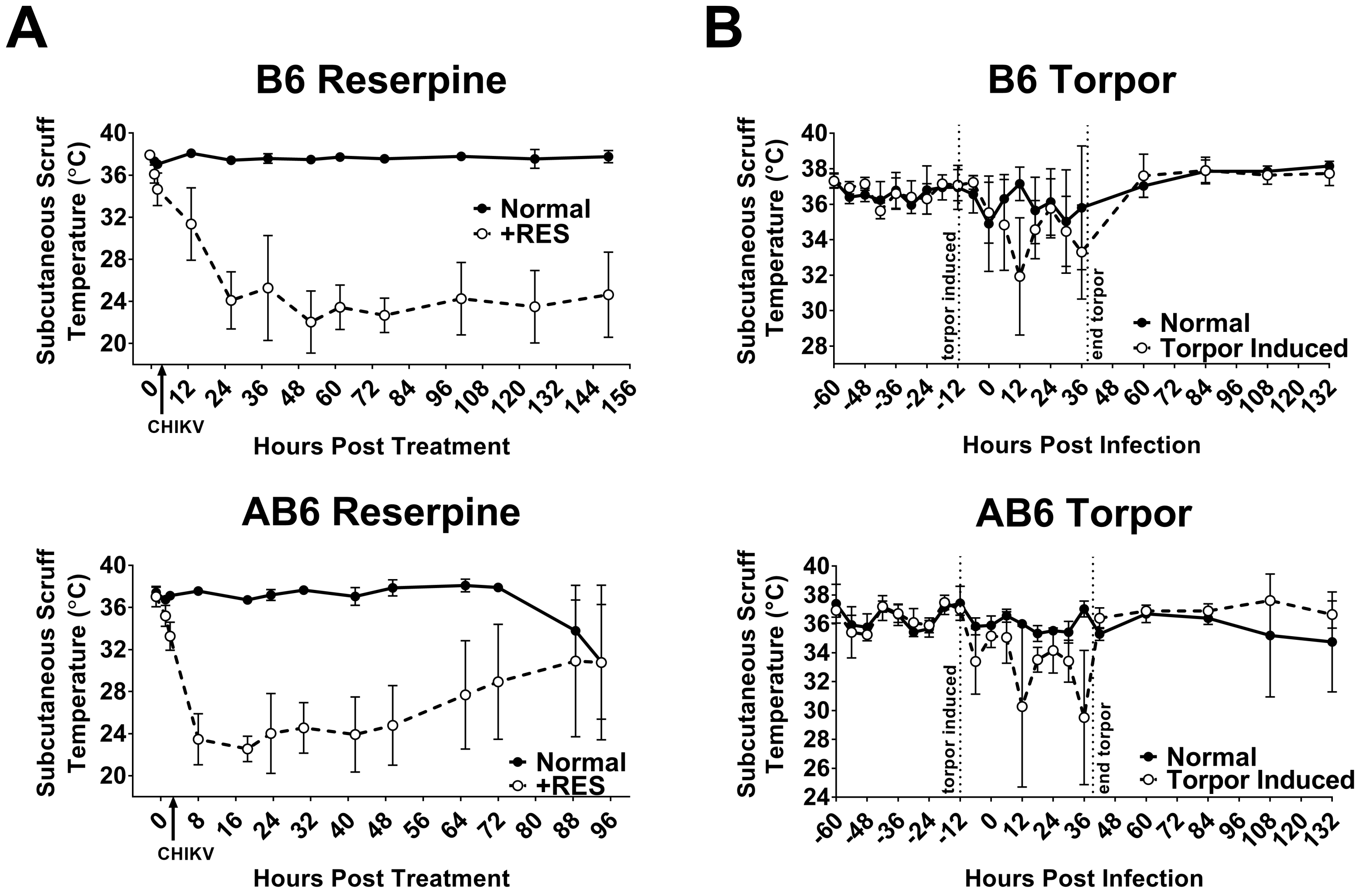

Figure S6: Torpor and reserpine treatment reduce mouse core temperature. Related to Figure 6.
Mice were implanted subcutaneously in the scruff with temperature transponders (BMDS) prior to administration of reserpine (**A**) or induction of metabolic torpor (**B**) as described in the Materials and Methods. Temperature was monitored regularly throughout the course of all experiments, and graphs represent the typical temperature profiles of reserpine-treated or torpid animals versus normal.
