# Supplementary material for: The Efficacy of the Interferon Alpha/Beta Response versus Arboviruses Is Temperature Dependent
Source: mBio. 2018 Apr 24;9(2):e00535-18. doi: 10.1128/mBio.00535-18 (PMC5915735; doi:10.1128/mBio.00535-18)
Supplement: TABLE S1 [file mbo002183831st1.pdf]

Table S1: Primers used for qRT-PCR		
Gene Name	Sense (5' → 3')	Antisense (5' → 3')
Primate *STAT1*	CACGCACACAAAAGTGATGA	ACATGTTCAGCTGGTCCACA
Primate *IFIT1*	TCTCAGAGGAGCCTGGCTAAG	CCACACTGTATTTGGTGTCTAGG
Primate *ISG20*	CTCCTGCACAAGAGCATCCA	CGTTGCCCTCGCATCTTC
Primate *ISG15*	CACCGTGTTCATGAATCTGC	CTTTATTTCCGGCCCTTGAT
Murine *Stat1*	TTCAGAGCTCCTTCGTGGT	TCGACTCTTGCAATTTCACC
Murine *Ifit1*	TGGCTCACATAGAGCAGGA	AGTTTCCTCCAAGCAAAGGA
Murine *Gbp2*	TGAGAAGGGTGACAACCAGA	AGCTCCGTCACATAGTGCAG
Murine *Isg20*	GGCACTGAGACAGGGCTT	CCATGGATGTTCACAATGCT
Murine *Il6*	TTCCATCCAGTTGCCTTCTT	CAGAATTGCCATTGCACAAC
Murine *Tlr2*	ACAACTTACCGAAACCTCAGAC	ACCCCAGAAGCATCACATG
Murine *Irf5*	CACCTCAGCCGTACAAGATC	GCCTGGTAGCATTCTCTGG
Murine *Ccl2*	GTCCCTGTCATGCTTCTGG	GCTCTCCAGCCTACTCATTG
Murine *Relb*	GCTGTACTTGCTCTGTGACA	TGGCGTTTTGAACACAATGG
Murine *Cxcl10*	TCAGCACCATGAACCCAAG	CTATGGCCCTCATTCTCACTG
Murine *Nfkbiz*	GGAATAAGAGCCTGGTAGACAC	AAGAGGCGAATGAGTTCCAG
Murine *Tnfaip3*	ACAGGACTTTGCTACGACAC	CTGAGGATGTTGCTGAGGAC
Murine Gamma Actin Intron #3	ACAGAACGCAAGCAGAAACG	TGGCATTTCCTCCCTGAAGC
Murine *Ifnb*	GAACATTCGGAAATGTCAGG	ACTGTCTGCTGGTGGATGTC
Murine *Ifna4*	CTGCTGGCTGTGAGGAAATA	GAAGACAGGGCTCTCCAGAC
18S rRNA	CGCCGCTAGAGGTGAATTCT	CGAACCTCCGACTTTCGTTCT
