# Supplementary material for: The Efficacy of the Interferon Alpha/Beta Response versus Arboviruses Is Temperature Dependent
Source: mBio. 2018 Apr 24;9(2):e00535-18. doi: 10.1128/mBio.00535-18 (PMC5915735; doi:10.1128/mBio.00535-18)
Supplement: TABLE S2 [file mbo002183831st2.pdf]

Table S2: List of NanoString Target Genes and Probe Locations		
Gene Name	*Accession Number*	*Probe Position*
β-Actin	NM_007393.3	42-141
γ-Actin	ENSMUST00000071555.2	1090-1189
ADAR1	NM_001146296.1	24-123
AKT1	NM_001165894.1	899-998
AKT2	NM_001110208.1	2505-2604
ATF-1	NM_007497.3	1217-1316
CCL2	NM_011333.3	416-515
CCL5	NM_013653.1	166-265
IKKα	NM_001162410.1	223-322
CIRP	NM_007705.2	203-302
CREB1	NM_001037726.1	2735-2834
CREBBP	NM_001025432.1	3771-3870
CSE1L	NM_023565.3	907-1006
CXCL1	NM_008176.1	561-660
CXCL10	NM_021274.1	116-215
eEF2	NM_007907.2	1520-1619
eEF2K	NM_007908.3	1167-1266
PKR	NM_011163.4	407-506
PERK	NM_010121.3	503-602
eIF2α	NM_026114.3	666-765
eIF3α	NM_010123.3	755-854
eIF4A1	NM_144958.4	1006-1105
eIF4E	NM_007917.3	697-796
eIF4EBP1	NM_007918.3	555-654
eIF4EBP2	NM_010124.2	236-335
eIF4G1	NM_145941.3	1981-2080
p300	NM_177821.6	4306-4405
c-fos	NM_010234.2	1331-1430
GAPDH	NM_008084.2	216-315
GBP2	NM_010260.1	1997-2096
GBP1	NM_010259.2	677-776
HSP90ab1	NM_008302.3	1355-1454
HSP70	NM_010478.2	2470-2569
IFI27	NM_026790.2	167-266
IFI44	NM_133871.2	991-1090
IFI47	NM_008330.1	710-809
IFIT1	NM_008331.2	891-990
IFIT2	NM_008332.2	231-330
IFIT3	NM_010501.1	1291-1390
IFITM1	NM_001112715.1	413-512
IFNα4	NM_010504.2	262-361
IFNAR1	NM_010508.1	1196-1295
IFNAR2	NM_001110498.1	726-825
IFNβ	NM_010510.1	336-435
IKKβ	NM_010546.2	499-598
IKKε	NM_019777.3	619-718

IKKγ	NM_178590.2	526-625
IL6	NM_031168.1	41-140
IRAK1	NM_008363.2	952-1051
IRAK4	NM_029926.5	251-350
IRF1	NM_008390.1	366-465
IRF2	NM_008391.2	441-540
IRF3	NM_016849.4	527-626
IRF5	NM_001252382.1	491-590
IRF7	NM_016850.2	706-805
IRF9	NM_008394.2	1406-1505
ISG15	NM_015783.1	396-495
ISG20	NM_020583.5	553-652
JAK1	NM_146145.2	4081-4180
c-jun	NM_010591.2	2213-2312
Importin α1	NM_008465.5	2671-2770
Importin β1	NM_008379.3	5136-5235
MEK	NM_008927.3	1696-1795
MAP2K3	NM_008928.4	1116-1215
MAP2K6	NM_011943.2	321-420
TAK1	NM_009316.1	823-922
ERK2	NM_011949.3	1211-1310
p38α	NM_011951.2	1421-1520
ERK1	NM_011952.2	826-925
MCM5	NM_008566.2	2610-2709
MKNK1	NM_021461.4	636-735
MKNK2	NM_021462.3	2877-2976
mTOR	NM_020009.2	2433-2532
MX1	NM_010846.1	2486-2585
MX2	NM_013606.1	2096-2195
MyD88	NM_010851.2	1596-1695
IκBα	NM_010907.1	941-1040
Nfkbiz	NM_030612.1	1306-1405
OAS1a	NM_145211.2	472-571
OAS2	NM_145227.3	1149-1248
PABP	NM_008774.3	863-962
PAP	NM_011112.3	4117-4216
PIAS1	NM_019663.3	1291-1390
PIAS3	NM_001165949.1	1137-1236
PI3K p100α	NM_008839.1	1256-1355
PI3K p85	NM_001024955.1	5665-5764
POLR2A	NM_001291068.1	2769-2868
PKCδ	NM_011103.2	1266-1365
PSMB9	NM_013585.2	541-640
PTPN1	NM_011201.3	510-609
SHP-2	NM_011202.3	3711-3810
PTPN2	NM_001127177.1	1013-1112
SHP-1	NM_013545.2	1692-1791
Ran	NM_009391.3	1756-1855
RBM3	NM_001166409.1	537-636

cRel	NM_009044.2	1291-1390
RelA	NM_009045.4	646-745
RelB	NM_009046.2	2014-2113
18S rRNA	NR_003278.3	1019-1118
RnaseL	NM_011882.2	2387-2486
RPS6	NM_009096.3	1196-1295
S6K1	NM_028259.4	1079-1178
Viperin	NM_021384.4	341-440
SOCS1	NM_009896.2	1021-1120
SOCS3	NM_007707.2	586-685
STAT1	NM_009283.3	1591-1690
STAT2	NM_019963.1	363-462
STAT3	NM_213659.2	2131-2230
TBK1	NM_019786.4	441-540
TBP	NM_013684.3	71-170
TRIF	NM_174989.4	2160-2259
TRAM	NM_173394.2	1244-1343
TIRAP	NM_001177845.1	1109-1208
TLR2	NM_011905.2	256-355
TLR4	NM_021297.2	2511-2610
TNFaIP3	NM_009397.2	233-332
TRADD	NM_001033161.2	563-662
TRAF3	NM_011632.3	885-984
TRAF6	NM_009424.2	981-1080
β-Tubulin	NM_009451.3	1820-1919
Tyk2	NM_001205312.1	1533-1632
USP18	NM_011909.2	1191-1290
Exportin 1	NM_134014.3	1905-2004
ZAP	NM_028864.2	2586-2685
